# A high-dimensional atlas of parvalbumin interneuron soma morphology in mouse visual and somatosensory cortex

**DOI:** 10.3389/fnins.2026.1848222

**Published:** 2026-06-10

**Authors:** Maheshwar Panday, Leanne Monteiro, Ahad Daudi, Kathryn M. Murphy

**Affiliations:** 1 McMaster Neuroscience Graduate Program, McMaster University, Hamilton, ON, Canada; 2 Department of Psychology, Neuroscience and Behaviour, McMaster University, Hamilton, ON, Canada

**Keywords:** cortical circuits, experience-dependent plasticity, laminar organization, parvalbumin interneurons, soma morphology, visual cortex (V1)

## Abstract

Parvalbumin-positive (PV+) inhibitory interneurons are central components of experience-dependent plasticity in the visual cortex (V1). Anatomically, they are distributed across cortical layers and are traditionally classified as basket, chandelier, or bipolar cells based on dendritic and axonal arborization patterns. In parallel, physiological, transcriptomic, connectomic, and multimodal studies have revealed substantial diversity within PV+ populations, raising the question of how this diversity is organized within cortical circuits. In contrast, light microscopy studies based on soma labeling have primarily quantified PV+ cells using size and density, and the potential of soma morphology to capture this diverse organization remains unclear. To address this, we developed a high-throughput, data-driven approach to quantify PV+ soma morphology in > 14,000 cells from mouse V1 and somatosensory cortex (S1). Using 97 morphological features combined with clustering, phenotyping, and laminar mapping, we identified structured diversity in PV+ somas. PV+ cells were organized into 13 morphological clusters along partially independent gradients of size and shape. Phenotyping identified four size and five shape categories that describe PV+ cell diversity across cortical areas. Mapping these categories onto cortical layers revealed a structured organization in which specific morphologies are enriched within distinct laminar compartments. This organization aligns with cortical architecture and suggests that PV+ interneuron morphology is systematically related to circuit structure. These findings demonstrate that substantial morphological information can be extracted from standard PV+ labeling approaches using quantitative analysis. Together, this work provides a high-dimensional atlas of PV+ interneuron soma morphology in mouse V1 and S1 and establishes a framework for linking cellular anatomy to circuit organization and experience-dependent plasticity.

## Introduction

Experience-dependent development of the visual cortex (V1) depends on the coordinated activity of excitatory and inhibitory neurons, with parvalbumin-positive (PV+) inhibitory interneurons playing a central role in regulating binocularity and ocular dominance plasticity (Kuhlman et al., 2010, 2013; Huang et al., 2024). Disruption of these inhibitory circuits is strongly implicated in the development of visual disorders such as amblyopia (Hensch and Quinlan, 2018). PV+ interneurons regulate the excitation–inhibition balance in V1 by providing fast-spiking, precisely timed inhibition onto pyramidal neurons (Hensch, 2005; Huang et al., 2024). Through this role, they contribute to Hebbian, homeostatic and spike-time-dependent forms of plasticity that fine-tune cortical circuits in response to visual experience (Maffei et al., 2006; Yazaki-Sugiyama et al., 2009; Runyan et al., 2010; Kuhlman et al., 2013; Nahmani and Turrigiano, 2014). They are also central to the timing of critical period plasticity, where their maturation governs the opening and closure of sensitive developmental windows (Pizzorusso et al., 2002; Sugiyama et al., 2008).

The functional properties of PV+ interneurons arise in part from their anatomical features. PV+ cells are classically described using dendritic and axonal arborization patterns, resulting in canonical morphological classes, such as basket, chandelier, and bipolar cells (Gonchar and Burkhalter, 1999; Wang et al., 2002; Karube et al., 2004; Ascoli et al., 2008). In contrast, anatomical studies using soma-labeling approaches, such as immunohistochemistry, have typically quantified PV+ cells by density and soma size, often treating them as a relatively uniform population.

At the same time, many studies have identified PV+ cell diversity through physiological, transcriptomic, and multimodal approaches. Physiological studies have highlighted diverse functional roles for PV+ interneurons (Markram et al., 2004; Kepecs and Fishell, 2014), while single-cell RNA sequencing has identified multiple PV transcriptomic types (Tasic et al., 2016). More recent multimodal studies integrating gene expression, morphology, and electrophysiology suggest that PV+ neurons occupy a continuous landscape of related phenotypes rather than forming discrete cell types (Gouwens et al., 2019; Scala et al., 2021). Together, this body of work reframes PV+ interneurons as a diverse and potentially continuous population, rather than a single canonical cell type.

In parallel, recent large-scale anatomical studies have begun to shift the focus from defining cell types to understanding how PV+ interneurons are embedded within cortical circuits. For example, connectomic analyses have characterized the organization of PV+ cells within the synaptic architecture of cortex, revealing structured patterns of connectivity that link inhibitory neurons to specific circuit motifs (Schneider-Mizell et al., 2025). At a finer structural scale, electron microscopy studies demonstrate that features of the soma and its immediate perisomatic environment contain substantial information about neuronal identity. For example, Elabbady et al. (2025) showed that inhibitory neuron subclasses can be identified using combinations of nuclear morphology, soma size, and postsynaptic structures.

However, despite these advances, it remains unclear whether PV+ soma morphology itself captures structured variation that aligns with laminar and circuit architecture. While many anatomical studies have quantified PV+ cell density and soma size (Whissell et al., 2015; Lingley et al., 2018; Bjerke et al., 2021), detailed characterization of soma shape has been largely overlooked. As a result, it is unknown whether soma morphology reflects meaningful organization within cortical layers and circuits that support experience-dependent plasticity.

Here, we address this gap by quantifying PV+ soma morphology. Rather than defining discrete cell types, we test whether soma morphology reveals structured variation within a continuous feature space and whether that structure is systematically organized across cortical layers and sensory areas. This approach allows us to ask whether PV+ soma morphology is linked to circuit architecture in V1 and somatosensory cortex (S1), providing a framework for understanding how inhibitory neuron structure contributes to experience-dependent cortical development.

We have quantified a large neuroanatomical dataset of PV+ cells in adult male mice obtained from the Allen-Institute-for-Brain-Science. (2004) mouse ISH database^1^ (Lein et al., 2007) to produce a database of soma morphologies in V1 and compared it with S1. We developed a high-dimensional analysis pipeline to quantify structured variation in PV+ soma morphologies. In addition, we integrated this soma-level information with an independent dataset of fully reconstructed PV+ interneurons to relate soma morphology to axonal and dendritic arborization patterns. Comparative analysis between V1 and S1 identified conserved and area-biased patterns of PV+ organization. Together, this dataset and analysis pipeline provide a reproducible, quantitative framework for light microscopy studies of PV+ interneurons, enabling detection of subtle structural differences relevant to cortical function and plasticity.

## Materials and methods

### V1 and S1 ISH images

All of the PV+ and laminar marker gene in situ hybridization (ISH) images of V1 and S1 were obtained from the Allen-Institute-for-Brain-Science. (2004) mouse ISH database (see text footnote 1) (Lein et al., 2007). This publicly available atlas was generated from male C57BL6 mice at postnatal day 56 (P56) following procedures that are documented in detail on the Allen Brain Map Community Forum.^2^ Briefly, the ISH and image processing were conducted by the Allen Brain Institute following standardized and automated procedures to ensure consistent, high-quality data. The procedures include producing probes for the genes examined, cryosectioning the tissue (25 μm), hybridizing with the probe using digoxigenin for colorimetric labeling of the cells, Nissl staining of adjacent sections, and image capture at high resolution (1.05 μm per pixel). The scanned images were processed to create a mask that identified neural cell-shaped objects, and then the integrated density of the ISH labeling was calculated for each object to create the expression images used in the current study.

The PV+ data used in the present study were obtained from 6 animals, and the quality of the ISH labeling was assessed to ensure comparable intensity across animals. Colourimetric ISH images from V1 and S1 for each animal were processed in FIJI (Schindelin et al., 2012), and the integrated density was measured for all cells in each processed image. Unpaired median differences in integrated density (calculated as cell area × mean gray value) across the six animals were assessed using estimation statistics with the dabestr package (version 0.3.0) in R (Ho et al., 2019). The labeling intensity of cells from one animal (Animal # 2293) was significantly lighter than that of the others, and was therefore excluded from further analysis (Supplementary Figure 1 and Supplementary Table 1).

### Datasets

Three datasets were constructed:

1. A dataset of ISH-labeled PV+ cells in V1 and S1
2. A dataset of ISH-labeled genes for 6 laminar marker genes in V1 and S1
3. A dataset of whole-filled PV+ cells

For the PV+ cells and laminar marker gene datasets, the colorimetric ISH and processed expression images were retrieved at full resolution (1.05 μm per pixel). Expression images were preprocessed by the Allen Institute for Brain Science to remove the background (black) and color-code cells by their integrated optical density reflecting the intensity of the ISH labeling (blue = minimum integrated density, red = high integrated density) (Ng et al., 2009). Cells in the expression images occasionally included small proximal neurite stubs; these were retained to preserve native soma geometry and because their contribution is limited relative to global shape descriptors. Count data for each dataset (number of cells by cortical layer) are provided in Supplementary Tables 2–4.

The PV+ cell dataset used 11 sagittal sections from each of the ISH and expression images spanning the medio-lateral extent of V1 and S1 for each of the 5 animals (11 sections × 2 image modalities × 5 animals × 2 brain areas). A sampling box from the pial surface to the white matter (567μm × 1,525 μm) was extracted from V1 and S1 for each of the ISH and expression images using Adobe Photoshop 2024.

The laminar marker gene dataset used ISH and corresponding expression images for these layer marker genes: L1—Ndnf, L2/3/4—Cux2, L4—Rorb, L5—Deptor, L6A—Foxp2, L6B—Ctgf. ISH and expression images used to prepare this dataset were also retrieved at full resolution (1 pixel = 1.05 μm). A sampling box with the same dimensions as described previously was extracted from each cortical area.

The whole-filled PV+ cells dataset was from the Allen Cell Types Database^3^ (Gouwens et al., 2019). The PV+ cells were extracted from 2 transgenic lines (Pvalb-IRES-Cre, Pvalb-T2A-FlpO) and were categorized as fully reconstructed cells and either aspiny or sparsely spiny. Cells were sampled from V1 (VISp) from layers 2/3 to 6A (no cells from layers 1 or 6B were filled). The database includes data from both sexes at P53   3 days, but the metadata does not specify the sex of the individual mice (Gouwens et al., 2019; Hodge et al., 2019).

Because the whole-filled PV+ dataset did not include cell-body expression images, we developed a workflow to extract cell bodies from the images. We downloaded images of the whole-filled cells (*n* = 36) and used the magic wand tool (tolerance parameters for each cell are reported in Supplementary Table 5) in Photoshop 2024 to capture the cell body. The somata were then pasted onto a “simulated cortex” to represent the laminar location of the filled cell. Nine of the 36 cells had a dark halo around the soma, so the levels tool was used to increase contrast, and the soma was then extracted with the magic wand. For these nine cells, the image histogram statistics are reported in Supplementary Table 18, following contrast adjustment with the levels tool.

### Image processing and cell morphology measurement

A custom image-processing pipeline was constructed in CellProfiler (Carpenter et al., 2006; Stirling et al., 2021) to prepare and quantify PV+ cell morphology (Supplementary material 1 and Supplementary Tables 6, 7). The pipeline converted the RGB expression images to grayscale (ColorToGray module, with equal weights of 1 given to each RGB channel), then binarized (Threshold module, Otsu’s two-class thresholding) and segmented the images (Watershed module, distance-based separation, footprint = 8, no downsampling) to identify particles for subsequent analysis. The processed images were used to measure 96 size and shape features (MeasureObjectSizeShape module), producing a data matrix in which each row represents a cell and each column contains spatial coordinates, metadata, and morphometric features of each segmented particle.

To remove artifacts and ensure that only neurons were analyzed, the dataset was filtered in RStudio to retain particles within a known neuronal size range (50–1,500 μm^2^) and circularity (0.2–1.0) for PV+ interneurons (Kooijmans et al., 2020). This step eliminated irregularly shaped particles and extremely large or small objects. Images identifying all analyzed cells were exported from CellProfiler and inspected by four independent observers to validate the pipeline. The final dataset included measurements from 14,274 PV+ interneurons.

#### Normalizing to a standard cortical thickness

To facilitate comparison of the laminar location of PV+ cells and the identification of cortical layers using marker genes, we standardized the y-dimension of all cells’ x-y coordinates. The distance from the pial surface to the bottom of layer 6B was measured at ten regularly spaced intervals across each image, and the y-coordinate for each cell was transformed such that 0 corresponds to the pial surface and 1 corresponds to the bottom of layer 6B. Cells with y-coordinates outside this range, either above the pial surface (y < 0) or below layer 6B (y > 1) were excluded.

#### Identifying cortical layers

We identified cortical layer boundaries using a set of 6 laminar marker genes (see above). For each marker gene, we derived laminar density profiles along the standardized cortical depth, using the density() function in R. To locate the boundary between adjacent cortical layers (e.g., Layers 4 and 5), we interpolated the density profiles with the approxfun() function and calculated the zero-crossings of their differences using uniroot.all() from the rootSolve package. Those intersection points represent a biologically grounded estimate of laminar boundaries in the standardized cortical thickness. Individual cells were subsequently assigned to layers according to their standardized cortical depth relative to the computed laminar boundaries (Supplementary Figure 2).

### Analysis of cell morphologies

#### Data preparation

Prior to analysis, cell metadata (cortical area, animal/sample ID, gene symbol, X/Y coordinates) were separated from the morphometric measurements. Redundant features that duplicated information about cell area (Central Moment 0_0, Spatial Moment 0_0, Inertia Tensor 0_1, and Inertia Tensor 1_0) were removed, producing a set of 96 size- and shape-related features. Additionally, we computed the aspect ratio as the ratio of the major to minor axis lengths. The final set of 97 features used for clustering is listed in Supplementary Table 8. Before clustering, the seven Hu Moment features (Hu Moments 0–6) were log-transformed to normalize their distributions and all 97 morphometric features were z-scored to place them on a common scale, and ensure each feature has equal potential to contribute to the downstream clustering solution.

#### Selecting the number of clusters

The optimal number of morphology clusters (k) was determined using the elbow method, which identifies the point at which further increases in k yield diminishing reductions in the within-cluster sum of squares (WCSS). WCSS values were calculated across a range of k-values using three independent functions in R: fviz_nbclust() from the factoextra package (Kassambara and Mundt, 2020), find_curve_elbow() from the pathviewr package (Baliga et al., 2023), and the elbow function from the elbow package (Casajus, 2020). An agreement between at least two methods was used to confirm the k-value for subsequent clustering.

#### Robust sparse K-means clustering

The PV+ cells were grouped into morphology clusters using the Robust Sparse K-Means Clustering (RSKC) algorithm (rskc() function from the RSKC package), an iterative and adaptive method that is robust to outliers (Kondo et al., 2016). A key component of RSKC is that it assigns weights to each feature, reflecting the relative contribution of each of the 97 size and shape measurements to cluster separation. This feature-weighting approach reduces the influence of correlated or weakly informative variables, preventing overrepresentation of feature groups that contain redundant measurements. Clustering was initialized with a trimming proportion (α = 0.1) to reduce the influence of outliers, and L1 bound (L1 = $\sqrt{97}$) to allow all 97 morphological features to contribute while still emphasizing the most informative ones. The number of clusters was set to *k* = 13 (per the elbow method), and the algorithm was run with 1000 random starts for stability.

#### Cluster validation

To assess morphological distinctness among clusters, we calculated composite size and shape scores by averaging the z-scores of the top-weighted size or shape features for each cell. These composite scores were assessed using a within-cluster design of estimation statistics, in which each morphology cluster’s composite size or shape score distribution was compared to the global distribution and median [dabestr package (version 0.3.0) in RStudio (Ho et al., 2019)]. Plotting these unpaired median differences showed a progression of distinct sizes and shapes across clusters.

#### Cluster visualization

The arrangement of the clusters was visualized using the density-preserving t-SNE (denSNE) algorithm (perplexity of 10, random seed 13579), which better retains the local density and global structure of data compared to standard t-SNE (Maaten and Hinton, 2008; Narayan et al., 2021). Before applying the denSNE algorithm, z-scored morphometric feature values were multiplied by their corresponding RSKC feature weights. This ensured that the visualization represented the clusters identified by RSKC (Balsor et al., 2021).

#### Cluster phenotyping to generate size and shape categories

Features selected by RSKC (i.e., non-zero-weighted features) were used to phenotype PV+ cell size and shape. For each morphology cluster, the median Z-scored value of each feature was computed to generate a cluster-level phenotype matrix (rows = features, columns = clusters), representing the characteristic feature profile of each cluster. Z-scores were calculated across all cells for each feature.

Size and shape features were analyzed separately to generate separate descriptive categories. Unsupervised hierarchical clustering (Ward.D2 linkage) was applied to each phenotype matrix to identify higher-order size and shape categories among clusters. The number of categories was determined using the elbow method based on within-cluster variance.

Cluster groups were then annotated using a supervised, criteria-based approach, in which feature profiles were interpreted alongside representative cell images to assign descriptive size and shape labels. This step provided biologically interpretable phenotypic categories while preserving the underlying data-driven structure. This phenotyping approach enabled the consolidation of morphology clusters into higher-order groups defined by shared anatomical size and shape characteristics.

#### Integrating filled cell data

Morphometric data for the whole-filled PV+ cells were prepared by analyzing the simulated cortex image in CellProfiler to measure the morphological features for each cell (Photoshop parameters for soma extraction are in Supplementary Table 5). These data were integrated with the ISH-labeled cells and analyzed with the cluster analysis pipeline described above.

Reconstructions of filled cells were done in RStudio using the neuroanatomy toolbox (nat) package (Bates et al., 2020) from the SWC files obtained for each cell from the Allen Cell Types Database. Additionally, the published annotations of dendritic and axonal arbor patterns for each cell were retrieved from the Allen Cell Types database (Gouwens et al., 2019) to compare the arbor structure and cell body morphology.

#### Laminar distribution of size and shape categories

The laminar locations of PV+ cells within each size and shape category were visualized to facilitate comparison of laminar patterns and analyzed separately for V1 and S1. For each cortical area, the expected laminar distribution was defined by the global proportion of PV+ cells across cortical layers, calculated from pooling all cells. Within each size/shape category, the observed proportion of cells in each layer was computed.

Laminar enrichment was assessed using a Monte Carlo resampling approach under a binomial null model. For each category-by-layer combination, a null distribution was generated by repeatedly sampling 10,000 times from a binomial distribution, with the number of trials corresponding to the total number of cells in the category and the probability defined by the global laminar proportion. Empirical one-sided *p*-values were calculated as the fraction of resampled proportions greater than or equal to the observed value, with a +1 continuity correction applied.

Multiple comparisons across all category-by-layer combinations were controlled using the Benjamini–Hochberg false discovery rate procedure. The magnitude of laminar enrichment was quantified using Cohen’s H. Categories with FDR-adjusted p-values < 0.05 were considered significantly enriched in specific cortical layers relative to the overall laminar composition.

## Results

### High-throughput quantification of PV+ soma morphology

**FIGURE 1:**
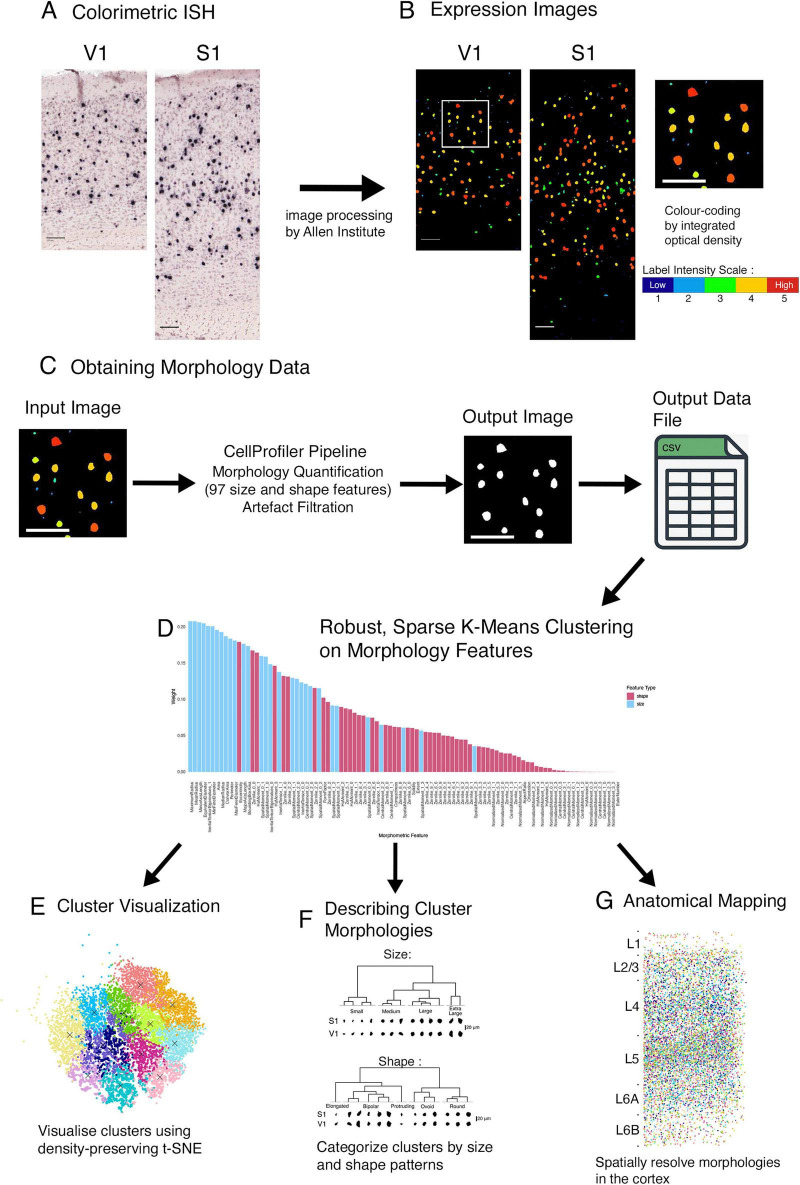
Workflow for high-throughput quantitative morphological analysis. **(A)** Colourimetric *in situ* hybridization (ISH) images from visual (V1) and somatosensory (S1) cortex labeled for the parvalbumin (PV) gene. PV+ cells are darkly labeled. Allen Mouse Brain Atlas, mouse.brain-map.org (Allen-Institute-for-Brain-Science., 2004, 2011). **(B)** ISH images were processed by the Allen Institute to generate Expression Images, in which cells are pseudocolored based on integrated density against a uniform black background. The inset shows a magnified region from the V1 sample. Allen Mouse Brain Atlas, mouse.brain-map.org. **(C)** Custom CellProfiler pipeline for image segmentation and morphological feature extraction. **(D)** Single-cell morphology data were analyzed using robust sparse k-means clustering. **(E)** Cluster organization was visualized using density-preserving t-SNE. **(F)** Morphological clusters were classified into size and shape categories. **(G)** Morphologies were mapped across cortical layers to quantify laminar distributions and area-specific biases between V1 and S1.

We developed a custom CellProfiler pipeline to enable high-throughput, quantitative analysis of PV+ interneuron morphology. Following data cleaning, the pipeline generated robust segmentation and feature extraction for 14,274 individual cells across 110 images from five animals, demonstrating reliable performance at scale. This approach produced high-dimensional morphological profiles for each cell while integrating animal- and specimen-level metadata within a unified framework. These data were subsequently used to identify groups of PV+ cells based on size and shape. Together, these components establish a scalable and reproducible approach for population-level morphological analysis, generating a large dataset of PV+ morphologies (Figure 1).

### Soma size alone does not distinguish PV+ cell populations across layers

**FIGURE 2:**
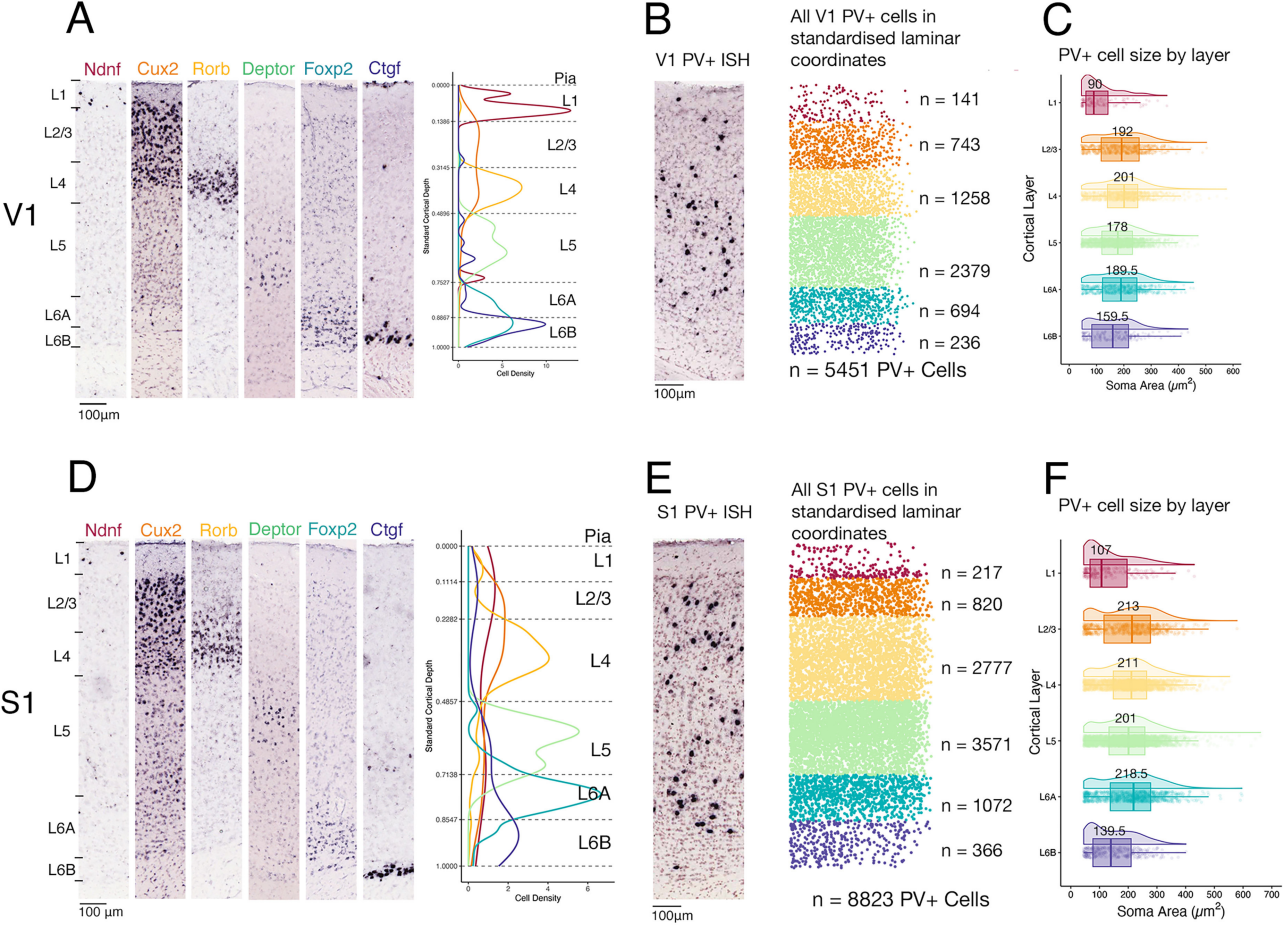
Layer mapping and soma size analysis in V1 and S1. **(A–C)** Data from V1, and **(D–F)** corresponding data from S1. **(A,D)** Cortical layer boundaries were identified using linear density profiles of laminar marker genes, with intersections defining putative laminar boundaries. **(B,E)** Representative colourimetric ISH images of PV+ cells are shown alongside maps of all PV+ cells, color-coded by their assigned cortical layers; cell counts for each layer are indicated. Scale bars = 100 μm. **(C,F)** Raincloud plots show the distribution of PV+ soma size across cortical layers, with median soma size (μm^2^) indicated for each layer and quartiles shown in boxplots beneath each distribution. The laminar distribution of PV+ cells in V1 was 2.5% in layer 1, 13.6% in layers 2/3, 23.1% in layer 4, 43.6% in layer 5, 12.7% in layer 6A, and 4.3% in layer 6B, whereas in S1 proportions were 2.5% in layer 1, 9.3% in layers 2/3, 31.5% in layer 4, 40.5% in layer 5, 12.2% in layer 6A, and 4.1% in layer 6B.

Classical anatomical analyses of PV+ interneurons often rely on soma size as a primary morphological metric for comparing cells across cortical layers. We began with this approach to assess whether soma size alone differentiates PV+ cells. The location of cortical layer boundaries was defined using laminar marker genes as described in the Methods (Figures 2A,D), and individual PV+ cells were assigned to layers based on their cortical depth relative to the boundaries (Figures 2B,E). Soma size was quantified using the CellProfiler area measurement (AreaShape_Area) (Figures 2C,F).

The laminar distribution of PV+ cells was broadly consistent with previous studies using immunohistochemistry (e.g., Park et al., 1999), with one notable difference: the increased sensitivity of ISH labeling revealed a small population of PV+ cells in layer 1. These layer 1 cells were small, consistent with reports that ISH can detect GABAergic neurons that may be below the detection threshold of immunohistochemical methods (Esclapez et al., 1993). Across cortical layers, PV+ cells were most abundant in layer 5, with smaller proportions distributed across layers 2/3, 4, and 6 in both V1 and S1 (Figures 2B,E).

Analysis of PV+ soma size across layers revealed broad, overlapping distributions in most layers in both V1 and S1, with smaller cells observed in layers 1 and 6B (Figures 2C,F). Estimation statistics (Supplementary Figure 3 and Supplementary Table 9) confirmed that PV+ cells in layers 1 and 6B are significantly smaller, while soma size does not distinguish PV+ cells among the remaining layers.

These results recapitulate the classical view of PV+ interneurons as a morphologically uniform population across cortical layers when assessed using soma size alone. However, soma size represents only a single dimension of morphology, motivating a more comprehensive analysis incorporating additional size and shape features.

### High-dimensional analysis reveals large variation in PV+ soma morphology

**FIGURE 3:**
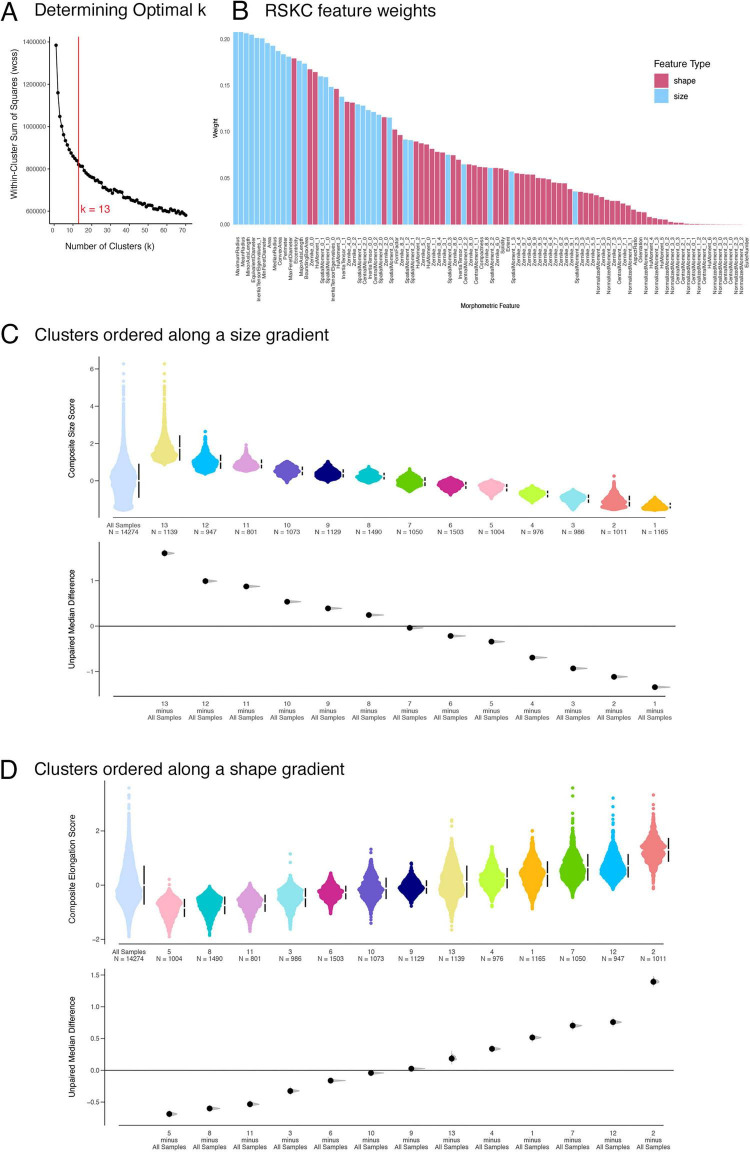
RSKC partitions PV+ interneurons into 13 morphological clusters. **(A)** The optimal number of clusters was identified using an elbow plot at *k* = 13. **(B)** Bar plot of morphological feature weights from RSKC, with size features shown in blue and shape features in red. **(C)** Cumming estimation plot for composite size scores across clusters. The upper panel shows swarm plots of composite size scores for each cluster, with the left-most distribution representing all cells in the dataset as a reference. The lower panel shows unpaired median differences relative to this reference, with the median indicated by a black dot and the 95% confidence interval represented by the bootstrapped distribution (shaded region). Cluster numbers reflect increasing median size from smallest (cluster 1) to largest (cluster 13). **(D)** Cumming estimation plot for composite elongation scores across clusters, presented as in **(C)**, with swarm plots in the upper panel and unpaired median differences with 95% confidence intervals in the lower panel.

**FIGURE 4:**
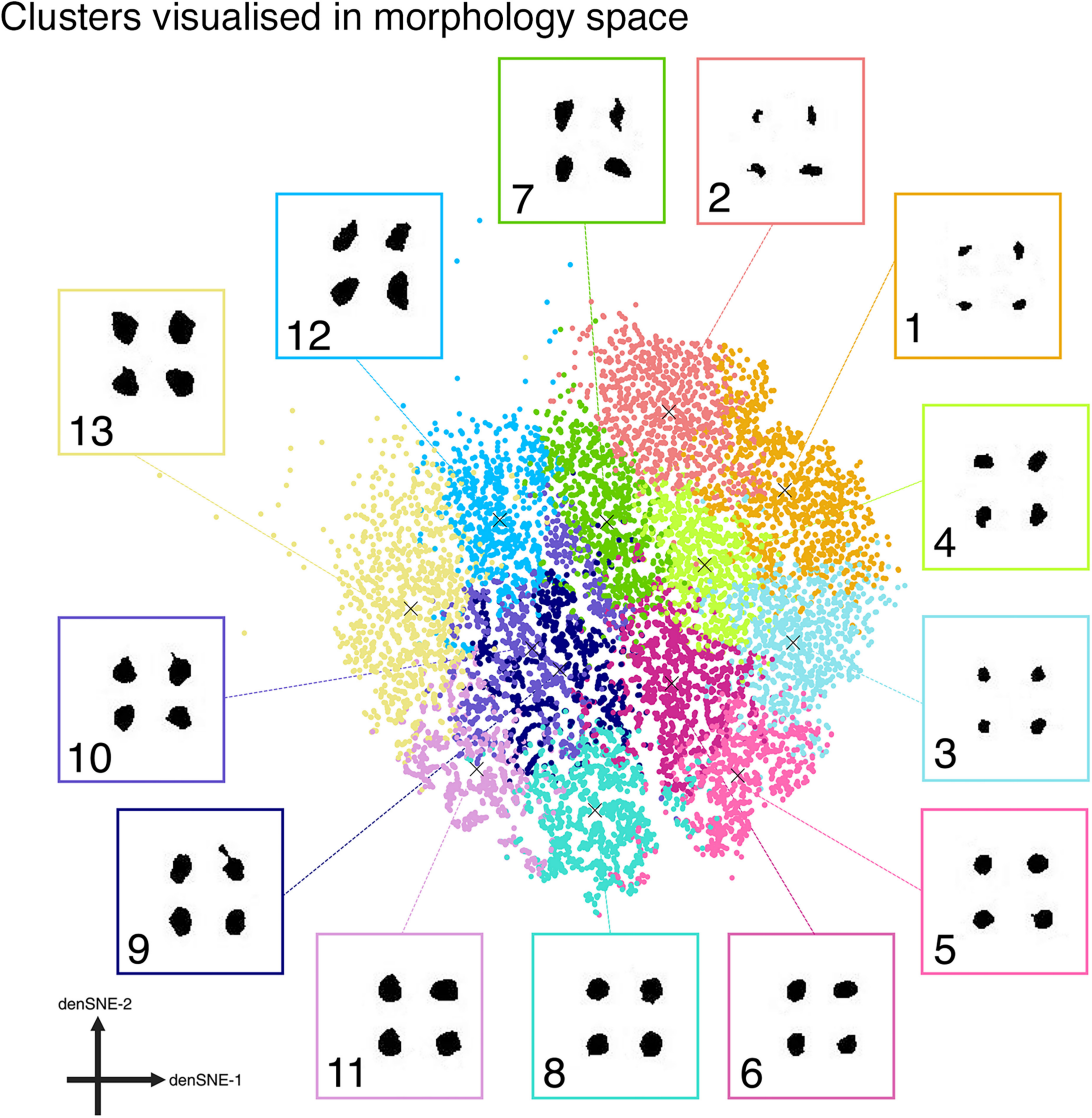
Visualization of PV+ morphology clusters using density-preserving t-SNE (denSNE). denSNE embedding of z-scored PV+ morphology data scaled by RSKC feature weights. Each point represents a single cell and is colored according to its assigned RSKC cluster. Cluster centroids are indicated by black crosses. Surrounding the embedding, four representative cells from each morphology cluster are shown to illustrate within-cluster morphological features. Cluster numbers (1–13) reflect increasing median soma size across clusters.

To capture morphological variation beyond soma size, we quantified PV+ interneuron morphology using a high-dimensional feature set encompassing multiple aspects of cell size and shape. Data-driven clustering of this feature space using RSKC identified 13 morphological groups (Figure 3A). Visualization using density-preserving t-SNE showed that these groups occupy separable regions of the morphological feature space, indicating non-random organization of PV+ soma morphology (Figure 4).

To rule out potential batch effects related to the animal of origin, we evaluated whether morphology clusters were driven by cells from individual animals. Visualization of the denSNE embedding, color-coded by animal, showed substantial overlap, with cells from all animals distributed across the full morphological feature space rather than forming animal-specific groupings (Supplementary Figure 4). Quantification of animal contributions within each cluster confirmed that all clusters contained cells from all animals in similar proportions. To further assess this, we calculated the normalized Shannon entropy (Pielou’s evenness) of animal representation within each cluster. Evenness scores were consistently high (0.953–0.999), indicating near-uniform contributions from all animals across clusters. Together, these analyses demonstrate that morphology clusters are not driven by batch effects between animals.

RSKC iteratively reweights features based on their contribution to cluster separation and analysis of those weights showed that both size and shape features drove the clustering (Figure 3B and Supplementary Table 8). While soma size features were among the most influential, multiple shape features, including metrics of elongation and circularity, also contributed substantially. A majority of features (64 of 97) received non-negligible weights and were retained for subsequent analyses, indicating that clustering was driven by a broad set of co-varying size and shape features rather than a small subset of variables.

To assess cluster separability, we evaluated composite size and shape scores across clusters using estimation statistics. Clusters were distributed along continuous gradients of both size and elongation, yet exhibited narrow, non-overlapping confidence intervals for unpaired median differences (Figures 3C,D, Supplementary Figure 4, and Supplementary Tables 11–14). Although PV+ soma morphology forms a continuous distribution, clustering revealed structured partitions along these axes, with adjacent clusters representing graded transitions rather than discrete boundaries. These results indicate that PV+ interneurons comprise reproducible morphological groups embedded within continuous size and shape dimensions.

### Phenotyping reveals distinct size and shape classes of PV+ soma morphology

**FIGURE 5:**
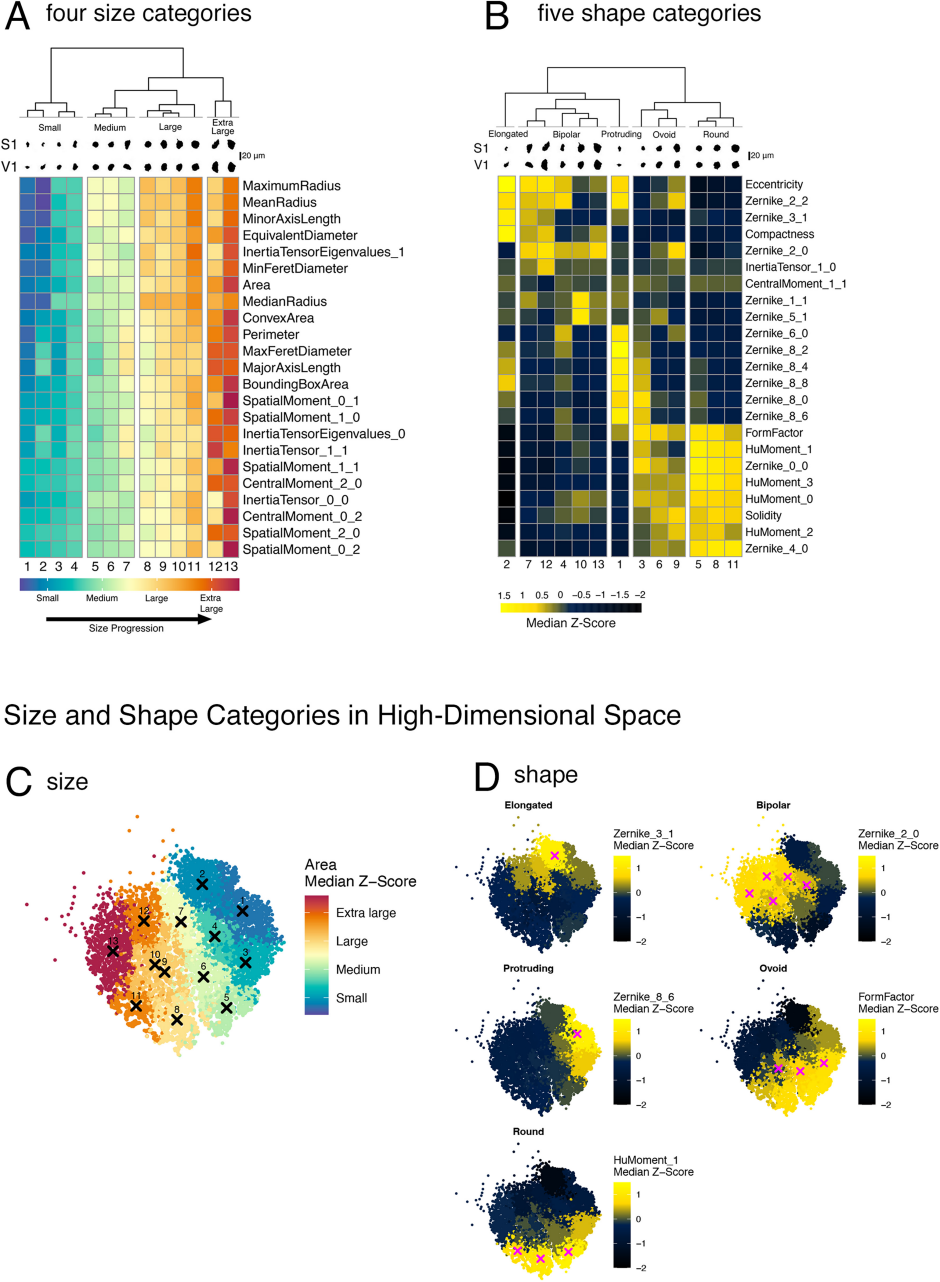
Phenotyping of PV+ morphology clusters reveals size and shape categories. **(A)** Heatmap of size phenotypes for each cluster, showing median z-scored values for the top 23 size-related features (rows) across the 13 morphology clusters (columns), ordered by unsupervised hierarchical clustering. This analysis identified four size categories: small, medium, large, and extra-large. Example cells from each cluster and cortical area are shown beneath the dendrogram. Scale bar = 20 μm. **(B)** Heatmap of shape phenotypes constructed similarly using median z-scored values for the top 23 shape-related features, revealing five shape categories: elongated, bipolar, protruding, ovoid, and round. **(C)** denSNE embedding from Figure 4 colored by size category, illustrating the distribution of size phenotypes across the morphological feature space, with cluster centroids indicated by black crosses. **(D)** denSNE embedding colored by shape category, with clusters assigned to each shape group highlighted and centroids marked by magenta crosses.

To translate the 13 morphology clusters into anatomically interpretable categories, we examined how they grouped along size or shape features. First, size features were used to identify four categories: small (clusters 1–4), medium (clusters 5–7), large (clusters 8–11), and extra-large (clusters 12–13) (Figure 5A). These categories captured the continuous gradient of soma size observed across clusters, providing a simplified framework for comparing groups of cells with similar size profiles.

Next, shape features were used to identify 5 categories that we annotated using common anatomical terms for soma shapes: elongated (cluster 2), bipolar (clusters 4, 7, 10, 12, 13), protruding (cluster 1), ovoid (clusters 3, 6, 9), and round (clusters 5, 8, 11) (Figure 5B). These categories reflected differences in elongation, symmetry, and contour complexity. For example, elongated and bipolar cells exhibit strong elongation with a defined major axis, whereas round cells display high circularity and lack directional structure. Ovoid cells exhibit intermediate features, combining aspects of circularity and elongation, whereas protruding cells exhibit irregular contours that are not captured by simple elongation metrics. Together, these shape categories map distinct regions of the morphological feature space onto interpretable anatomical descriptors (summary statistics for each size-shape category are provided in Supplementary Table 15).

To visualize how the size and shape phenotypes were represented in the morphological space, we color-coded the denSNE embedding using either the size or shape categories (Figures 5C,D). This revealed that soma size and shape vary along approximately orthogonal axes. A gradient of soma size extended along the horizontal axis, while shape variation, from round to elongated cells, was organized along the vertical axis. The 13 clusters tiled the intersection of these gradients across the denSNE.

**FIGURE 6:**
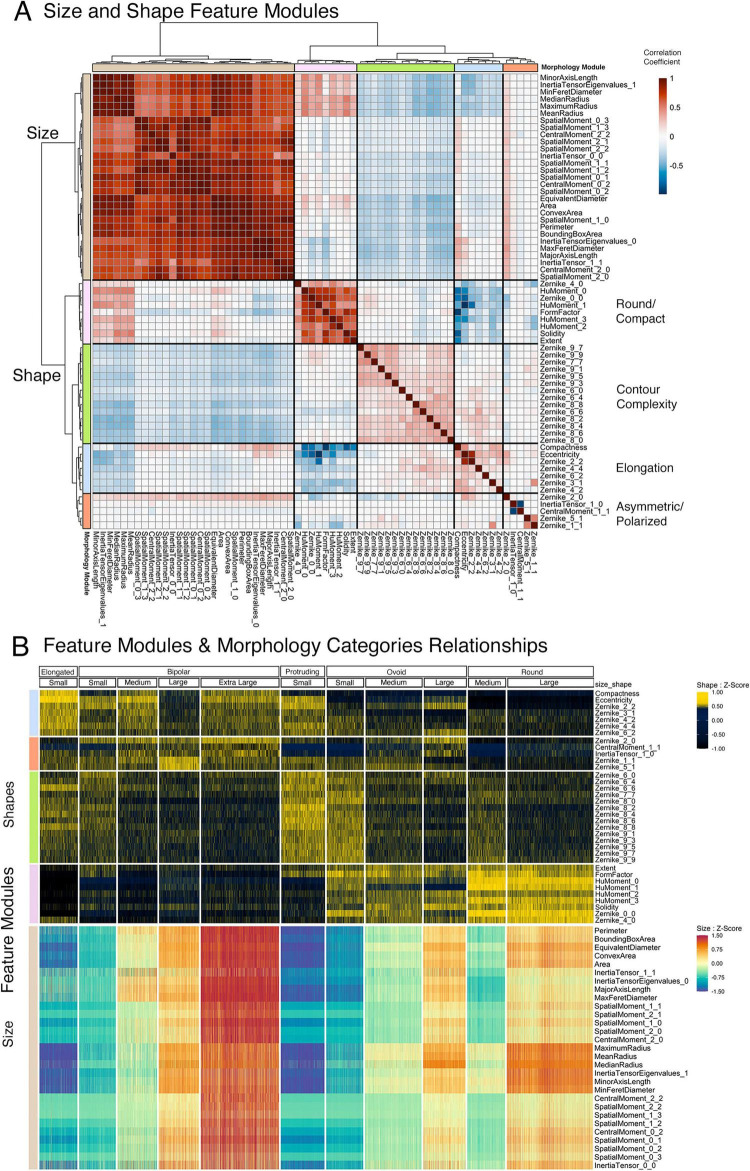
Feature covariance and module structure underlying PV+ soma phenotypes. **(A)** Correlation heatmap of all morphological features, showing the organization of feature covariation. Size-related features form a large module of strongly co-varying measurements, with subgroups of highly correlated size features. In contrast, shape-related features are organized into four smaller, less strongly correlated modules. Inspection of these modules indicates that they capture distinct aspects of soma morphology, including elongation, asymmetric/polarized structure, contour complexity, and round/compact geometry. **(B)** Heatmap showing the relationship between feature modules (rows) and morphology categories (columns; e.g., Elongated-Small). Cells within each category exhibit similar feature values, indicating that categories reflect consistent combinations of morphological features. Shape modules collectively define the major shape categories (Elongated, Bipolar, Protruding, Ovoid, and Round), with distinct patterns of feature values across modules. Elongated and Round categories are strongly distinguished by high values within individual shape modules, whereas Ovoid cells show intermediate values. In contrast, Bipolar and Protruding categories are defined by more distributed patterns, with moderate values across multiple shape modules. The size module values clearly separate the PV morphologies into Small, Medium, Large and ExtraLarge categories.

Since size and shape categories were not strictly coupled, with shape categories spanning multiple sizes and size categories including diverse shapes, we sought to understand how the various features contributed to this independence. We therefore analyzed the covariation among all morphological features. A correlation heatmap revealed that size features formed a large module of co-varying features, with subgroups of highly correlated size measurements (Figure 6A). In contrast, shape features were organized into four smaller, less strongly correlated modules. Examination of the features within each shape module showed that they quantified distinct attributes of PV+ somata, including elongation, asymmetric/polarized structure, contour complexity, and round/compact morphology (Table 1).

**TABLE 1:** Shape module features and interpretation.

Shape module	Features (high)	What the features capture	Morphological interpretation
Elongated	Eccentricity, Compactness (inverse FF), Zernike (2,2), (4,4)	Axis elongation, elliptical symmetry, orientation along a major axis	Elongated, cigar-shaped or bipolar somata with defined axis
Asymmetric/polarized	Zernike (1,1), (5,1), low Inertia Tensor (1,0), low Central Moment (1,1)	Off-center mass, directional asymmetry without strong elongation	Lopsided, skewed or bipolar somata; not elongated but not symmetric
Complex	High-order Zernike (6–9): (9,7), (9,9), (7,7), (9,1), (9,5), (9,3), (6,0), (6,4), (6,6), (8,8), (8,2), (8,4), (8,6), (8,0)	Fine-scale contour complexity, lobulation, irregular boundary structure	Irregular, lobulated, or protruding somata with non-smooth edges
Round/compact	HuMoment 0–3, Zernike (0,0), Form factor, Solidity, Extent	High radial symmetry, compactness, smooth boundary, fills bounding box	Large, round or ovoid somata with smooth contours; isotropic

Next, we examined how the size and shape feature modules related to the morphology categories assigned to each cell (e.g., Elongated-Small). A heatmap of feature modules (rows) and morphology categories (columns) revealed several key patterns (Figure 6B). Cells in a morphology category had similar values for each feature, indicating that categories reflect consistent combinations of morphological properties. The shape modules combined to define the major shape categories (Elongated, Bipolar, Protruding, Ovoid, and Round), with distinct patterns of feature values across modules. For example, individual shape modules strongly distinguished Elongated and Round categories based on high feature values (bright yellow), whereas Ovoid cells exhibited intermediate values. In contrast, the Bipolar and Protruding categories were defined by more distributed patterns, with moderate values across multiple distinct shape modules (Table 2).

**TABLE 2:** Linking somata shape categories with shape features and modules.

Shape category	Dominant feature pattern (high/low)	Shape modules engaged	Morphological description
Elongated	High eccentricity; high compactness (inverse FF); high Zernike (2,2), (4,4); low asymmetry and complexity	high Elongation	Strongly axis-oriented, cigar-shaped; smooth boundaries
Bipolar	Very high eccentricity; strong Zernike (2,2); moderate Zernike (4,4); low solidity/extent relative to size axis; low complexity	mild Elongation, mild Asymmetric / Polarized	Highly elongated with clear axis; often tapered ends; aligned geometry
Protruding	High Zernike (1,1), (5,1); high high-order Zernike (6–9); lower form factor/solidity; low-to-moderate eccentricity	high Elongation, high Complex	Irregular, lobulated, or bumpy somata; non-symmetric, non-elliptical
Ovoid	Moderately high form factor and solidity; moderate eccentricity; moderate Zernike (2,2), (4,4); low high-order Zernike	mild Round/Compact	Slightly elongated but smooth and compact; elliptical
Round	High form factor, solidity, extent; high HuMoment 0–3; high Zernike (0,0); low eccentricity, low high-order Zernike	high Round / Compact	Symmetric, isotropic, smooth somata; close to circular

The features in the size module were relatively homogeneous within each morphology category. However, some variations within the size module were visible as horizontal bands of features. These bands suggest that although the size features co-vary, there are subtle variatios in information that contributes to the structured separation among size categories. Together, this phenotyping framework provides an interpretable representation of PV+ soma diversity, linking high-dimensional morphological structure to biologically meaningful size and shape classes.

We next asked whether these morphological groups are conserved across sensory cortices or exhibit area-specific specialization.

### PV+ soma morphologies are conserved across V1 and S1 with area-biased abundance

Cells from both V1 and S1 were represented across all 13 morphology clusters, indicating that the overall repertoire of PV+ soma morphologies is largely conserved between sensory cortices. However, differences in relative abundance were observed across clusters. Clusters 3–6 were enriched in V1, whereas clusters 11–13 were enriched in S1 (Supplementary Figure 5 and Supplementary Table 16). These shifts reflect changes in the proportion of shared morphological classes rather than the presence of area-specific morphologies. Together, these findings indicate that PV+ interneurons exhibit a conserved set of soma morphologies across sensory cortices, with area-dependent variation in the relative representation of specific morphological groups.

### Laminar organization of PV+ soma morphologies reveals a shared structural logic with area-dependent modulation

**FIGURE 7:**
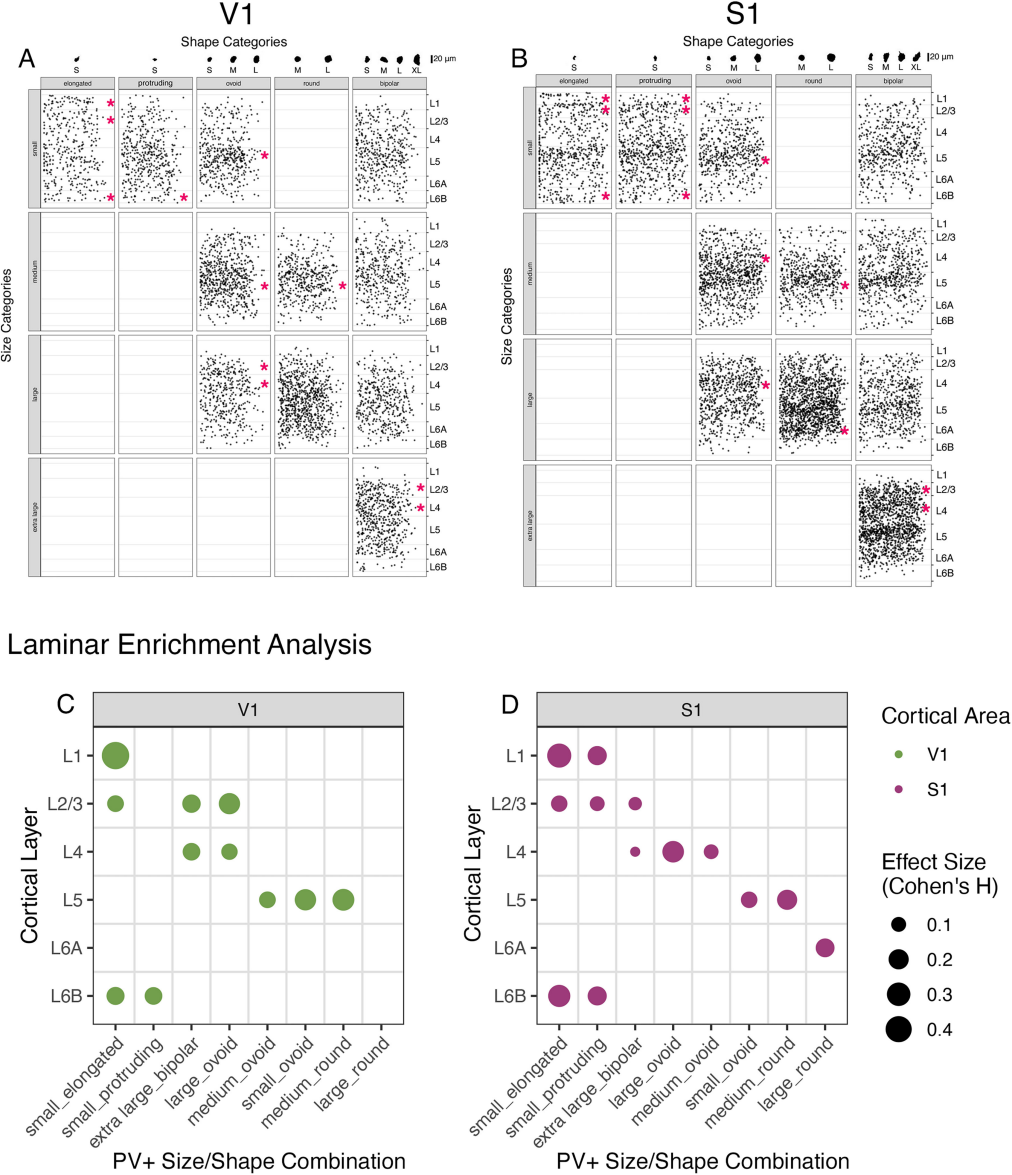
Laminar enrichment of PV+ cell morphologies in V1 and S1. Spatial distribution of PV+ interneurons across cortical layers for each size and shape category in V1 **(A)** and S1 **(B)**. Each point represents a single cell, showing that all morphology categories are broadly distributed across layers in both cortical areas. Example cells are shown above each shape category, illustrating the range of sizes represented within that category (scale bar = 20 μm). Asterisks (red *) indicate cortical layers in which specific morphology categories are significantly enriched (FDR-adjusted *p* < 0.05). **(C,D)** Summary of laminar enrichment patterns shown in **(A)**, presented as bubble charts for V1 **(C)** and S1 **(D)**. Bubble size represents the magnitude of enrichment (Cohen’s H), highlighting morphology-specific biases across cortical layers.

Mapping size–shape phenotypes onto cortical layers revealed that PV+ soma morphologies are not uniformly distributed, but instead exhibit laminar biases (Figures 7A,B). In both V1 and S1, morphologies characterized by higher circularity (ovoid and round) were preferentially localized to layer 5, whereas lower circularity morphologies were enriched outside layer 5 across size classes (Figures 7C,D and Supplementary Tables 17, 18). These findings indicate that morphological phenotype is systematically related to laminar position.

This relationship reflects the structured organization of morphological space. Size–shape combinations are not freely distributed, but occupy restricted regions defined by specific pairings. For example, protruding and elongated morphologies were confined to the small size class, whereas extra-large cells were exclusively bipolar. In contrast, ovoid and round morphologies were restricted to medium and large size classes. These constraints define distinct regions of morphological space that map onto specific laminar domains.

Despite this shared organizational framework, the strength and distribution of laminar biases differed between cortical areas. In both V1 and S1, ovoid and round morphologies were enriched in layer 5; however, in S1, these cells formed a dense intralaminar band, whereas in V1, they were more diffusely distributed within the layer. Lower circularity morphologies were enriched outside layer 5 in both areas, but again showed more spatially concentrated distributions in S1 compared to V1. Extra-large bipolar cells, while relatively sparse in V1, were more abundant in S1 and exhibited broader laminar distributions.

Together, these results demonstrate that PV+ interneuron morphology is linked to laminar organization via a shared structural principle whereby specific size–shape phenotypes preferentially occupy distinct cortical layers. This organizing logic is conserved across sensory cortices, but its expression is modulated in an area-dependent manner. Furthermore, given the role of PV+ interneurons in regulating cortical plasticity, this laminar organization supports the idea that inhibitory control may be structured in a layer-specific manner.

### Integration with filled cells links soma morphology to arbor organization

To relate soma morphology to arborization measures of neuronal structure, we integrated a set of 36 whole-filled PV+ interneurons from the Allen Cell Types dataset (Gouwens et al., 2019), which include detailed dendritic and axonal reconstructions. Soma morphology was extracted from these cells and analyzed within the same high-dimensional framework as the ISH-derived dataset.

**FIGURE 8:**
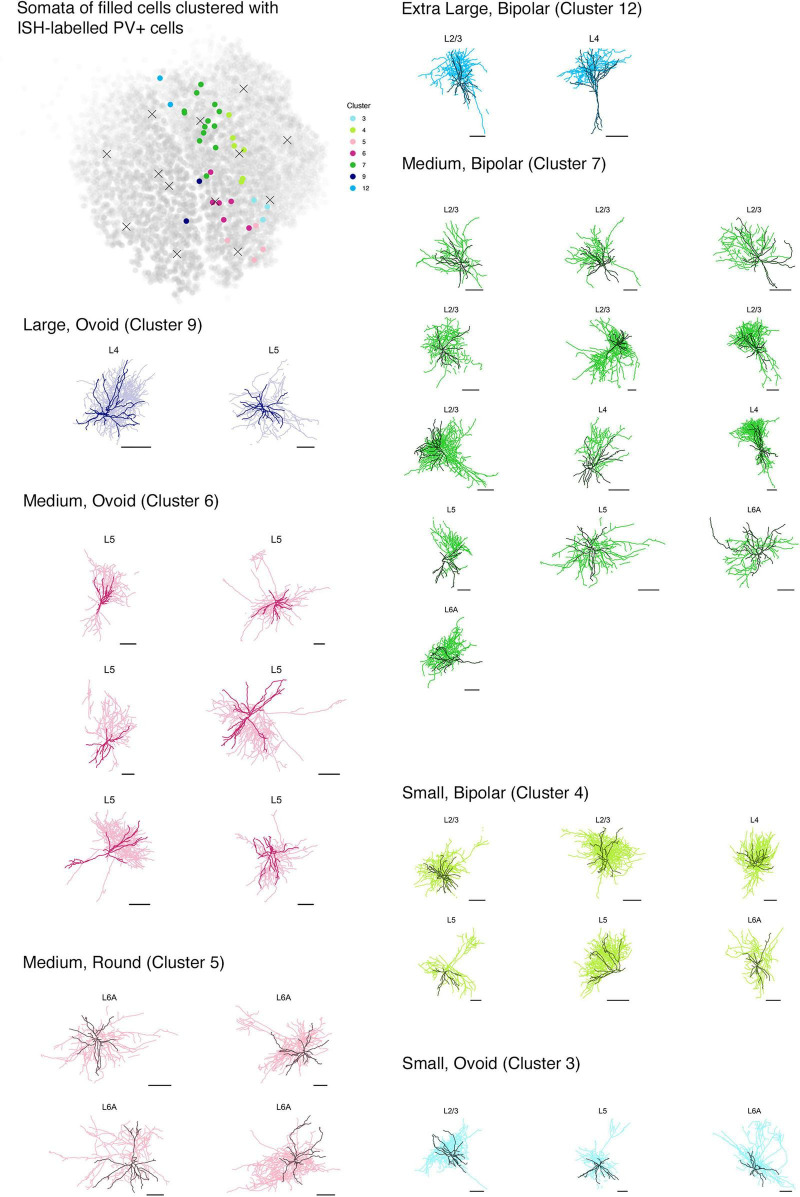
Filled cell integrations identify emerging associations between soma morphology and arbor structure. denSNE embedding plot showing the integrated PV+ morphology dataset. ISH-labeled PV+ interneurons are shown in gray, and whole-filled cells are colored according to their RSKC clusters. Cluster numbers reflect median size progression. Surrounding the denSNE plot are reconstructions of the dendritic and axonal arbors of the filled cells. All scale bars = 100 μm. In all reconstructions, dendrites are colored in darker hues and axons in lighter hues. Above each reconstruction is the cortical layer in which the cell’s soma is located.

Filled cells mapped onto the established morphological space and distributed across multiple clusters, rather than forming a distinct group (Figure 8). Successful patching, filling, and full axonal and dendritic reconstruction likely favor cells within a restricted range of soma sizes and morphologies. Smaller somata may be less amenable to patching, whereas very large or extensively arborized cells may be less likely to yield complete reconstructions. Consistent with this, the filled cells occupied the middle region of the denSNE while still spanning bipolar, ovoid, and round shape categories. Their positions followed the same size and shape gradients observed in the ISH-labeled cells, indicating that the identified morphological structure generalizes across datasets and imaging modalities.

Within this shared morphological space, soma shape showed emerging associations with arbor organization. Cells with strongly elongated, bipolar somata tended to exhibit dense, radially oriented arbors (Figure 8 clusters 4, 7, 12), whereas more circular or round somata were associated with broader, more heterogeneous arbor patterns, including greater tangential spread (Figure 7 cluster 5). Ovoid cells, representing intermediate morphologies, showed mixed projection patterns (Figure 8, clusters 3, 6, 9), suggesting that soma shape is related to graded variation in arbor organization rather than to discrete wiring classes.

Together, these findings indicate that soma morphology provides a useful axis for integrating structural datasets and reveals potential links between cell shape and circuit organization. While limited in sample size, this analysis suggests that high-dimensional soma features capture aspects of neuronal architecture that extend beyond the soma itself.

## Discussion

In this study, we developed a high-throughput, data-driven approach to quantify PV+ soma morphology and applied it to a large population of cells in mouse V1 and S1. We found that PV+ somas exhibit structured diversity that can be resolved into 13 morphological groups defined by combinations of size and shape features. These groups are embedded within continuous gradients of size and shape, which we translated into interpretable phenotypic categories. Those morphological groups were not randomly distributed; instead, specific size–shape phenotypes exhibited systematic laminar enrichment, revealing a shared organizing logic linking soma morphology to cortical architecture. This organization was conserved across sensory cortices, with area-dependent differences in the relative abundance of specific morphologies. Together, these findings establish a scalable framework for quantifying PV+ soma morphology and linking it to the laminar organization of sensory cortices that regulate experience-dependent plasticity.

### Structured diversity of PV+ soma morphology

Classical anatomical categorizations of PV+ interneurons have focused on dendritic and axonal arborization patterns, leading to classifications such as basket, chandelier, and bipolar cells (Markram et al., 2004; Ascoli et al., 2008). In parallel, a large body of work has established substantial diversity among PV+ interneurons using physiological, transcriptomic, connectomic, and multimodal approaches (Tasic et al., 2016; Gouwens et al., 2019; Scala et al., 2021; Schneider-Mizell et al., 2025). Many previous studies have quantified PV+ cells in the cortex using soma size and density, whereas soma shape has been less frequently incorporated into large-scale analyses. Recent electron microscopy work has demonstrated that features of the soma and its immediate perisomatic environment contain substantial information about interneuron identity (Elabbady et al., 2025). However, these approaches are not readily scalable to large populations using standard light microscopy methods such as ISH or IHC.

The current study provides a high-throughput, population-scale framework to quantify PV+ soma morphology using standard ISH data, enabling systematic analysis across cortical areas. We show that PV+ soma morphology comprises a high-dimensional combination of size and shape features organized within a structured morphological space. Rather than defining discrete cell types, this framework captures reproducible patterns within a continuous size–shape landscape, thereby linking soma morphology to circuit organization. In this context, soma morphology provides a complementary anatomical dimension that can be measured at scale. This approach leverages widely available ISH datasets, enabling reproducible and scalable analysis of neuronal morphology without requiring full reconstructions.

### Phenotyping reveals independent axes of size and shape

To interpret this variation in PV morphology, we implemented a phenotyping step that categorized morphological clusters into classes associated with anatomical descriptors. The clusters were grouped into four size categories and five shape categories, in which soma size reflects graded variation and shape captures combinations of elongation, circularity, and contour complexity. Importantly, size and shape are not coupled: shape spans multiple size classes, and size includes multiple shapes, indicating that these features represent approximately independent axes of soma variation. This phenotyping framework reveals an interpretable anatomical organization of PV morphology, demonstrating that soma diversity is structured rather than random. Furthermore, this approach can be applied to other cell-types and brain regions to characterize morphological variations linked to circuit organization.

### Laminar organization of PV+ soma morphology reflects circuit architecture

Mapping morphological phenotypes onto cortical layers revealed that PV+ soma morphologies are not uniformly distributed, but instead exhibit distinct laminar biases. In both V1 and S1, specific size–shape phenotypes show preferential localization to particular layers, indicating that PV morphological diversity is systematically organized within cortical circuits.

The laminar distributions observed here were broadly consistent with previous immunohistochemical studies of PV+ interneurons (Blümcke et al., 1990; Brederode et al., 1991; Park et al., 1999; Xu et al., 2010), with one notable difference: ISH labeling revealed a population of PV+ cells in layer 1. Those cells are consistent with reports of PV expression in early development (Hendrickson et al., 1991) and multipolar bursting PV+ neurons found along the border between layers 1 and 2/3 (Blatow et al., 2003). The layer 1 cells in our study were small and may have been below the detection threshold of immunohistochemical studies, possibly reflecting low levels of PV protein expression. In addition, cortical layer boundaries in the present study were defined using the density of laminar marker genes rather than cytoarchitectural criteria. As a result, some cells near the border between layers 1 and 2 may have been assigned to layer 1, whereas an anatomist using Nissl staining might classify them as belonging to layer 2. Importantly, these factors likely contribute to minor differences in laminar proportions without altering the overall pattern of layer-specific organization observed across cortical areas.

We observed a systematic enrichment of specific size–shape phenotypes across cortical layers, suggesting that soma morphology may reflect layer-specific circuit roles. The small, elongated cells enriched in layers 1 and 6B may support subtle, spatially constrained inhibitory modulation in regions dominated by distal dendrites and long-range modulatory inputs. In contrast, extra-large bipolar cells enriched in layers 2/3 and 4 are well positioned to provide strong inhibitory output and may participate in columnar integration by receiving dense axosomatic inputs that contribute to gain control and feedforward inhibitory control (Porter et al., 2001; Cardin et al., 2009; Hofer et al., 2011; Atallah et al., 2012; Lee et al., 2012; Wilson et al., 2012). Large ovoid cells, which are also enriched in these layers, are consistent with both fast-spiking and multipolar-bursting PV interneurons and may help stabilize network activity by maintaining the E/I balance (Hensch, 2005) or generating rhythmic oscillatory activity (Blatow et al., 2003), respectively. In layer 5, circular morphologies are enriched and may provide stable, symmetric inhibitory control over large projection neurons, supporting reliable output signaling.

Together, the layer-specific enrichment of distinct morphological phenotypes suggests that soma size and shape may be adapted to the differing circuit demands across cortical layers, with smaller, elongated cells predominating in layers associated with modulatory and distal dendritic processing, and larger, more circular cells enriched in layers that support strong perisomatic inhibition and output regulation. These relationships suggest that PV soma diversity is aligned with known gradients of cortical computation, from modulatory and integrative processing in superficial and deep layers to feedforward control and output regulation in middle and deep layers.

### Soma morphology aligns with inhibitory circuit geometry

To link soma morphology to circuit organization, we integrated whole-filled PV+ interneurons into the morphological framework (Gouwens et al., 2019). All filled cells were classified as basket cells, allowing us to examine variation within a canonical PV subtype. Rather than defining distinct anatomical classes, soma morphology aligned with known cortical circuit motifs. Bipolar morphologies, enriched in layers 2/3 and 4, were associated with vertically oriented arborization patterns, consistent with roles in columnar integration and feedforward inhibitory control across layers. In contrast, round morphologies observed in layer 6a were associated with broader, tangentially distributed projections, suggesting a role in lateral inhibition and in regulating spatial integration within deep-layer output circuits. Ovoid morphologies, which spanned multiple layers and size classes, were associated with mixed projection patterns, consistent with more flexible roles in integrating local and interlaminar activity.

These relationships suggest that soma shape is aligned with the spatial geometry of inhibitory signaling within cortical circuits, with bipolar, round, and ovoid morphologies associated with vertically oriented, laterally distributed, and mixed patterns of inhibition, respectively. In this framework, soma size may scale the strength of inhibitory output, while shape captures how inhibition is deployed across space and layers. These findings indicate that morphological diversity within PV basket cells is aligned with fundamental principles of cortical circuit organization rather than representing discrete cell-type categories.

### Conserved architecture with area-specific specialization across V1 and S1

Although the overall repertoire of PV+ soma morphologies was conserved across V1 and S1, we observed subtle but consistent differences in the relative abundance of specific phenotypes. In V1, layer 2/3 was enriched for large ovoid morphologies, whereas in S1, layer 6A showed enrichment for large round cells. These differences suggest that while a common set of morphological phenotypes is shared across sensory cortices, their relative representation is tuned to the specific circuit demands of each area. Layer 2/3 in V1 is strongly engaged in intracortical integration and experience-dependent plasticity, and the enrichment of larger ovoid cells may reflect a need for stable, high-conductance inhibitory control within these recurrent networks (Ranson, 2017). In contrast, layer 6A in S1 contains corticothalamic projection neurons, and the enrichment of large, circular morphologies may support symmetric and robust inhibitory control over both cortical and thalamic firing (Folkard et al., 2026). Together, these findings indicate that PV+ interneuron morphology reflects a conserved structural framework that is modulated in an area-dependent manner to support distinct functional demands across cortical circuits.

### PV interneuron morphology and experience-dependent plasticity

PV+ interneurons are central regulators of experience-dependent plasticity. Their development governs critical period timing, controls the E/I balance, and shapes the maturation of ocular dominance (Hensch, 2005; Hensch and Quinlan, 2018). Moreover, PV+ cells contribute to a range of receptive field properties, including orientation, direction, spatial frequency, and contrast selectivity, which form the foundation of visual perception (Runyan et al., 2010; Rosario et al., 2025). Abnormal early visual experience, such as monocular deprivation, leads to a rapid reduction in PV-mediated inhibition followed by a rebound and strengthening of inhibitory signaling. These transient changes in PV+ activity are necessary for shifts in ocular dominance toward the open eye (Kuhlman et al., 2013). Over development, the role of PV+ inhibition transitions from supporting plasticity to stabilizing cortical circuits as neurons become ensheathed by perineuronal nets (Pizzorusso et al., 2002).

While PV interneurons are known to be diverse, our findings show that this diversity is organized within laminar circuits in a structured manner. For example, plasticity-relevant layers 2/3 and 4 are enriched for large and extra-large PV+ morphologies that would have the metabolic capacity to support feedforward gating and recurrent circuit dynamics underlying experience-dependent changes in visual processing. These observations support the idea that plasticity is not mediated by a uniform inhibitory population, but instead by morphologically distinct PV subgroups embedded within specific cortical circuits. Our atlas provides a framework for testing whether particular PV morphologies are selectively altered by abnormal visual experience and whether such changes are layer- or morphology-specific. More broadly, this approach enables population-level quantitative analysis of inhibitory circuit plasticity.

### Limitations

There are several limitations to consider. First, the biological scope of this study was limited to young adult male mice (P56) at the end of the critical period, and therefore does not address how PV+ soma morphology changes during development or whether there are differences between the sexes. Second, our analysis focused on soma morphology. Although the large ISH dataset enabled high-throughput, consistent sampling across a substantial cell population, it does not capture the full complexity of PV interneuron structure, which is also defined by dendritic and axonal arborization patterns. While the inclusion of a small set of whole-filled cells provided initial evidence linking soma morphology to arbor organization, this dataset was not sufficient to fully resolve these relationships. Third, soma morphology was quantified from 2D ISH images, whereas neuronal somata are 3D structures, and cell orientation or partial sectioning may influence measurements from individual cells. However, all sections were sampled in the same sagittal orientation and analyzed within a common anatomical framework, and the large number of cells should reduce the influence of random projection effects on population-level patterns. If truncation were the primary driver of small or irregular morphologies, those cells would be expected to be broadly distributed across layers. Instead, small morphology classes showed distinct laminar enrichments, suggesting that the observed patterns reflect biological organization rather than sectioning artifacts alone. Fourth, Third, the links to cortical circuits and plasticity are inferred from soma morphology and laminar position rather than directly measured. These interpretations are grounded in established circuit principles, but require validation using physiological and connectivity-based approaches. Notably, our findings generate testable hypotheses, such as whether subtle differences in soma size and shape distinguish fast-spiking and multipolar-bursting PV+ cells in layers 2/3. Despite these limitations, this study provides a robust, population-level analysis of PV+ soma morphology in mouse sensory cortex. Importantly, the pipeline developed here is scalable and can be readily extended to examine developmental trajectories, experience-dependent plasticity, and cross-species comparisons.

## Conclusion

The core contributions of this study are threefold. First, we mapped PV+ interneuron soma morphology at scale using a high-throughput, quantitative approach. Second, we found that PV+ soma morphology exhibits structured diversity organized along continuous, partially independent gradients of size and shape. Third, this structure is aligned with laminar variations in cortical circuits, such that soma morphology reflects the geometry of inhibitory signaling in V1 and S1. This morphological diversity provides additional support for the idea that experience-dependent plasticity is mediated by distinct PV subgroups embedded within specific circuits, thereby linking morphology-specific changes to plasticity and amblyopia. The framework developed here is scalable and enables future studies to examine how PV+ and other neuron morphologies are shaped across development, sensory experience, and species, offering a quantitative foundation for understanding cortical circuit organization and function.

## Data Availability

The datasets and code presented in this study can be found in the online repository 10.17605/OSF.IO/8F2NR. The names of the repository/repositories and accession number(s) can be found in the article/Supplementary material.

## Footnotes

1
https://mouse.brain-map.org/

2
https://community.brain-map.org/t/documentation-allen-mouse-brain-atlas/2846

3
https://celltypes.brain-map.org/data
