# Supplementary material for: A high-dimensional atlas of parvalbumin interneuron soma morphology in mouse visual and somatosensory cortex
Source: Front Neurosci. 2026 Jun 10;20:1848222. doi: 10.3389/fnins.2026.1848222 (PMC13290952; doi:10.3389/fnins.2026.1848222)
Supplement: Supplementary file 2 [file Data_Sheet_1.pdf]

Supplementary Tables

Table S1 – *Integrated Density Quality Control Across Animals***.** Median integrated density is reported for each animal, with unpaired median differences relative to animal # 2293. Bootstrapped 95% confidence intervals are shown. Integrated density is defined as the product of cell size and mean gray value, reflecting label intensity. Supports Supplementary Figures 1B-C.

Table S2 - *PV+ Cell Counts by Layer and Cortical Area.* Counts of PV+ cells organized by cortical layer (rows) and cortical area (columns)

Table S3 - *Laminar Marker Gene Cell Counts by Layer***.** Counts of cells labeled for laminar marker genes via ISH, organized by cortical layer.

Table S4 - *Filled Cell Counts by Layer and Driver Line* . Counts of filled cells tabulated by cortical layer (rows) and diver line (columns).

Table S5 - *Image Parameters for Soma Extraction from Filled Cells.* For each cell, the magic wand tool was used to extract the soma, the specific tolerance, sampling strategy, and wand parameters are recorded. For cells with a levels adjustment, to control the contrast, the image histogram statistics are recorded following contrast adjustment.

Table S6- *CellProfiler Experiment Files for V1 Image Analysis.* Experiment file from CellProfiler specifying the modules and parameter settings used to analyse PV+ ISH expression images from V1.

Table S7 - *CellProfiler Experiment Files for S1 Image Analysis.* Experiment file from CellProfiler specifying the modules and parameter settings used to analyze PV+ ISH expression images from S1.

Table S8 – *RSKC feature weights and feature classifications.* Feature weights from RSKC are indicated, along with feature classification as a size or shape parameter.

Table S9 – *Estimation Statistics for PV+ cell soma area by cortical area in V1 and S1.* Median soma size (AreaShape_Area, μm^2) for each cortical area × layer combination with unpaired median differences relative to the global PV+ soma size distribution. Bootstrapped 95% confidence intervals are shown. Supports Supplementary Figure 3.

Table S10 – *Estimation statistics for composite size scores for 13 PV+ morphology clusters.* Median composite size scores for 13 PV+ morphology clusters are reported, with unpaired median differences relative to the global distribution. Bootstrapped 95% confidence intervals are indicated. Composite size is defined as the mean of z-scored, top-weighted size features for each cell. (Figure 5A, heatmap row labels). Supplements Figure 4C.

Table S11 – *Estimation statistics for composite elongation across morphology clusters.* Median composite elongation scores for 13 PV+ morphology clusters are reported, with unpaired median differences relative to the global difference. Bootstrapped 95% confidence intervals are indicated. Composite elongation is defined as the mean of the z-scored Zernike 2_0, 2_2 , 3_1 moments and Compactness for each cell. Supplements figure 4D.

Table S12 – *Estimation statistics for composite circularity across morphology clusters.* Median composite scores for 13 PV+ morphology clusters are reported, with unpaired median

differences relative to the global distribution. Bootstrapped 95% confidence intervals are shown. Composite circularity is defined as the mean of the z-scored Zernike 0_0, and 4_0 moments, Hu moments 0-3, Form Factor and Solidity values for each cell. Supports Supplementary Figure 4A.

Table S13 – *Estimation statistics for composite concavity across morphology clusters.* Median composite concavity scores for 13 PV+ morphology clusters are reported with unpaired median differences relative to the global distribution. Bootstrapped 95% confidence intervals are shown. Composite concavity is defined as the median of the z-scored Zernike 1_1, Zernike 5_1, Inertia Tensor (1_0) and Central Moment (1_1) features for each cell. Supports Supplementary Figure 4B.

Table S14 – *Estimation statistics for composite protrusion across morphology clusters.* Median composite protrusion scores for 13 PV+ morphology clusters are reported, with unpaired median differences relative to the global distribution. Bootstrapped 95% confidence intervals are shown. Composite protrusion is defined as the mean of the z-scored higher order Zernike moments (6_0, 8_0, 8_2, 8_4, 8_6, 8_8). Supports Supplementary Figure 4C.

Table S15 - *Descriptive statistics for PV+ morphology size and shape categories.* Means, medians, 95% confidence intervals and quartiles for the top 23 size and shape features are reported for each morphology class. These statistics provide quantitative benchmarks for the descriptive terms used to annotate the size and shape categories.

Table S16 - *Area bias statistics for 13 PV+ morphology clusters.* Chi square statistics and cell proportions by cortical area reported for all 13 PV+ morphology clusters. P-values were computed using the chi square test for goodness of fit and corrected for multiple comparisons

using the false discovery rate procedure (FDR). Clusters with significant area biases are indicated by FDR-corrected $p < 0.05$.

Table S17- *Laminar enrichment statistics for PV+ Morphology classes in V1.* For each PV+ morphology and cortical layer, observed and expected proportions are reported. Effect sizes are quantified using Cohen's H. Empirical p-values are provided alongside false discovery rate (FDR) - corrected p-values. Significant enrichments (FDR corrected $p<0.05$) are indicated with asterisks. P-values were estimated using a Monte Carlo simulation under a binomial null model.

Table S18 - *Laminar enrichment statistics for PV+ Morphology classes in S1.* As in table S12 observed and expected proportions are reported for each morphology class across layers, along with effect sizes (Cohen's H), empirical p-values and FDR-corrected p-values. Significant enrichments (FDR-corrected $p<0.05$) are indicated with asterisks. P-values were estimated using a Monte Carlo simulation under a binomial null model.

Supplementary Materials

Supplementary Material 1 - *Protocol for image analysis and morphology quantification.* This document provides step-by-step instructions for image analysis using CellProfiler. It includes guidance on image retrieval with links to the Jupyter Notebook published by the Allen Institute for Brain Science as part of their software development kit (allensdk). This was used for programmatic access to mouse ISH data. The protocol covers file-naming conventions for handling metadata, image processing, morphology quantification and evaluating filtering thresholds for data cleaning.
