# Supplementary material for: A high-dimensional atlas of parvalbumin interneuron soma morphology in mouse visual and somatosensory cortex
Source: Front Neurosci. 2026 Jun 10;20:1848222. doi: 10.3389/fnins.2026.1848222 (PMC13290952; doi:10.3389/fnins.2026.1848222)

Supplementary Figure Legends

Supplementary Figure 1 – *Assessment of label quality among animals.* (A) Representative samples of colourimetric ISH from each animal in V1(i) and S1(ii) . Each of the samples show darkly labelled PV+ interneurons compared to the lighter background. Importantly, labelling is lighter in animal 2293. In all images, scale bars = 100 µm. (B) Swarm plots of integrated density values from each cell in the representative sample images. The left-most distribution shows cells from animal 2293 which were used as a reference. (C) Unpaired median differences for the remaining five animals, compared to animal 2293 are shown in the estimation plot. The unpaired median difference is shown as black dot, and the bootstrapped 95% confidence interval is shown as a vertical black bar. The bootstrapped distribution is shown as the shaded region.

Supplementary Figure 2 – *An unsupervised approach to map the positions of cortical layers.* (A) Colourimetric ISH for laminar marker genes in V1, showing clear, laminarly restricted patterns of gene expression along the cortical depth. (B) Laminar boundaries in V1 were identified as the calculated intersections between pairs of laminar profiles mapping pairs of adjacent layers. (C) ISH for laminar marker genes in S1. (D) Unsupervised layer boundary calculations in S1, where the intersections between adjacent laminar profiles are taken as putative layer boundaries. In A and C, scale bars = 100 µm.

Supplementary Figure 3 – *Size analysis reveals limited morphological diversity across PV+ interneurons.* Cumming estimation plot of cell size by cortical layer for each cortical area. The upper axes show swarm plots of cell area distributions for each cortical layer by area. S1 cells are in purple, and V1 cells are in green. Cortical depth progresses from left to right. Darker swarms

indicate deeper cortical layers. The left-most swarm shows the distribution of cell sizes from all cells in the dataset (all layers, from both areas), and is used as a reference. The lower axes show estimation plots, for the unpaired median differences, shown as black dots. The vertical black line corresponds to the bootstrapped 95% confidence interval, and the bootstrapped distribution is shown in the shaded region.

Supplementary Figure 4 – *Quantifying animal-specific distributions across clusters.* (A) denSNE plots of cells from individual animals, with each animal plotted separately. (B) Stacked bar plots showing the proportion of cells from each animal within each cluster. Colours correspond to the animal IDs in panel A. (C) denSNE plot of all cells, coloured by the normalized Shannon entropy of each cluster. A value of 0 indicates that a cluster contains cells from a single animal, whereas a value of 1 indicates an equal contribution from all animals.

Supplementary Figure 5 – *Additional estimation statistics on composite shape properties for 13 PV+ morphology clusters.* Cumming estimation plots for (A) composite circularity, (B) composite concavity, (C) composite protrusion. In all plots, the upper axes show the swarm plots for the composite shape property scores for each of the 13 morphology clusters. The left-most swarm in each of the plots represents the composite shape score distributions for the entire dataset (all PV+ cells from all clusters combined) and is used as a reference. The unpaired median differences are shown in the estimation plots, as black dots on the lower axes. The bootstrapped 95% confidence intervals are shown as the vertical black bars and the bootstrapped distributions are shown as the shaded regions. In all plots, cluster numbers 1-13 reflect the progression of median cell size.

Supplementary Figure 6 - *Area biases of PV+ morphology clusters.* (A) Stacked proportion barplot showing the global distribution of PV+ cells from S1 and V1 (61.91% S1 : 38.19% V1, all cells combined). (B) Stacked bar plots showing for each of the 13 morphology clusters the proportion of PV+ interneurons originating from S1 and V1. Asterisks indicate clusters where a cortical area is significantly more represented relative to the global distribution (chi square test for goodness of fit, FDR–corrected $p < 0.05$).

Assessing label quality among animals

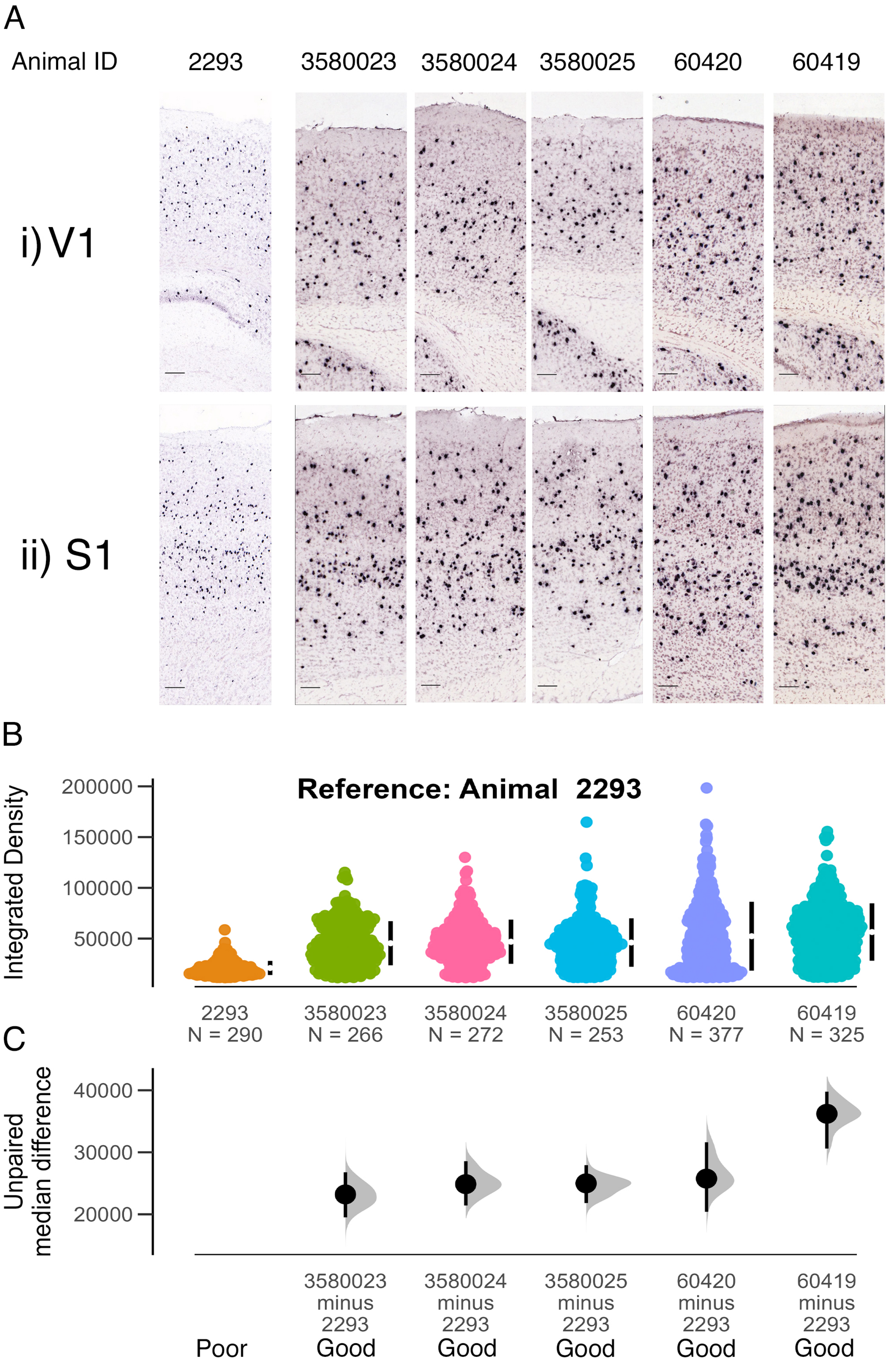

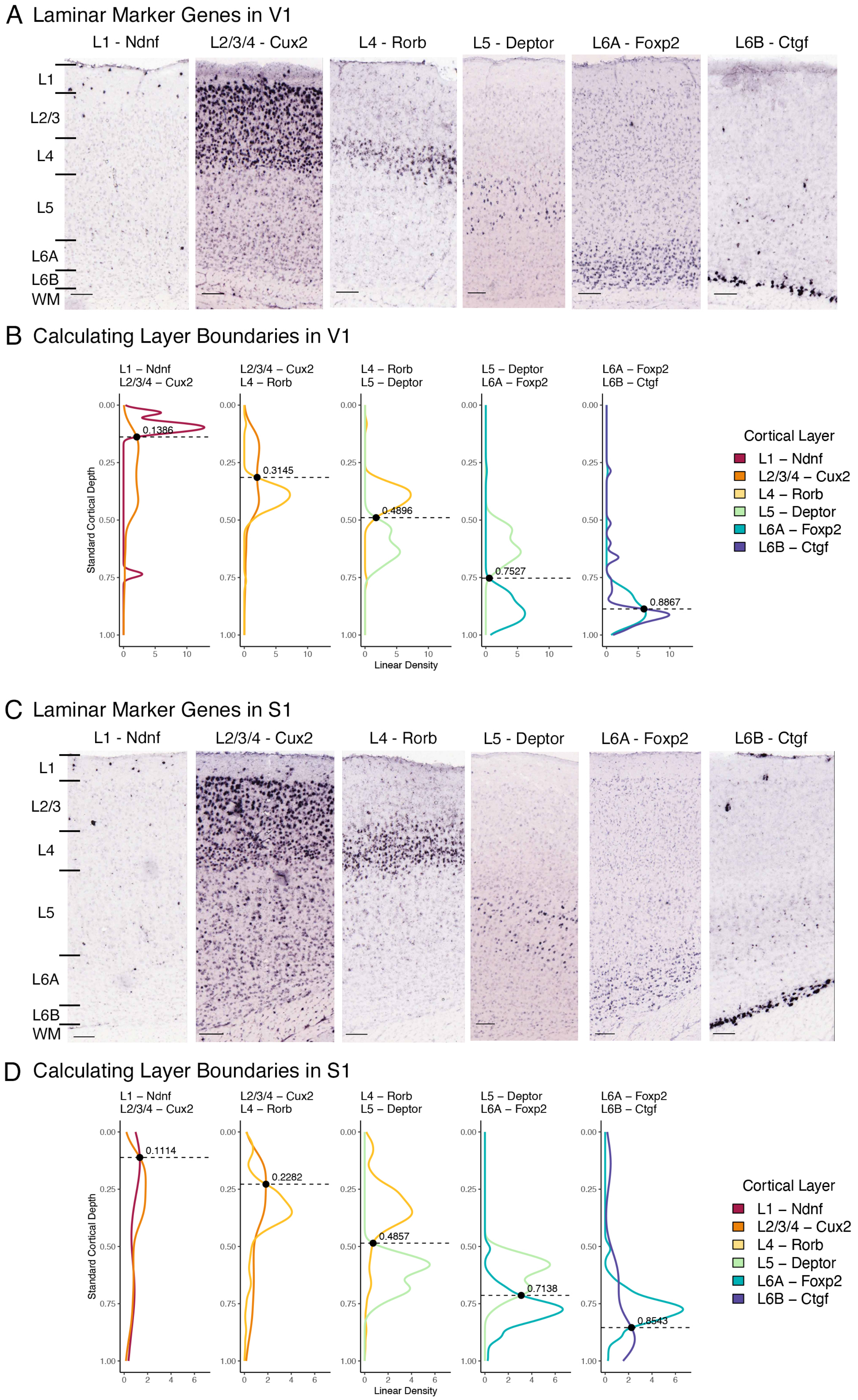

A Laminar Marker Genes in V1
L1 - Ndnf
L2/3/4 - Cux2
L4 - Rorb
L5 - Deptor
L6A - Foxp2
L6B - Ctgf
L1
L2/3
L4
L5
L6A
L6B
WM
B Calculating Layer Boundaries in V1
L1 – Ndnf
L2/3/4 – Cux2
L4 – Rorb
L5 – Deptor
L6A – Foxp2
L6B – Ctgf
Standard Cortical Depth
Linear Density
0.1386
0.3145
0.4896
0.7527
0.8867
Cortical Layer
C Laminar Marker Genes in S1
D Calculating Layer Boundaries in S1
0.1114
0.2282
0.4857
0.7138
0.8543

Supplementary Figure 3
Comparing Cell Size Across Cortical Areas and Layers

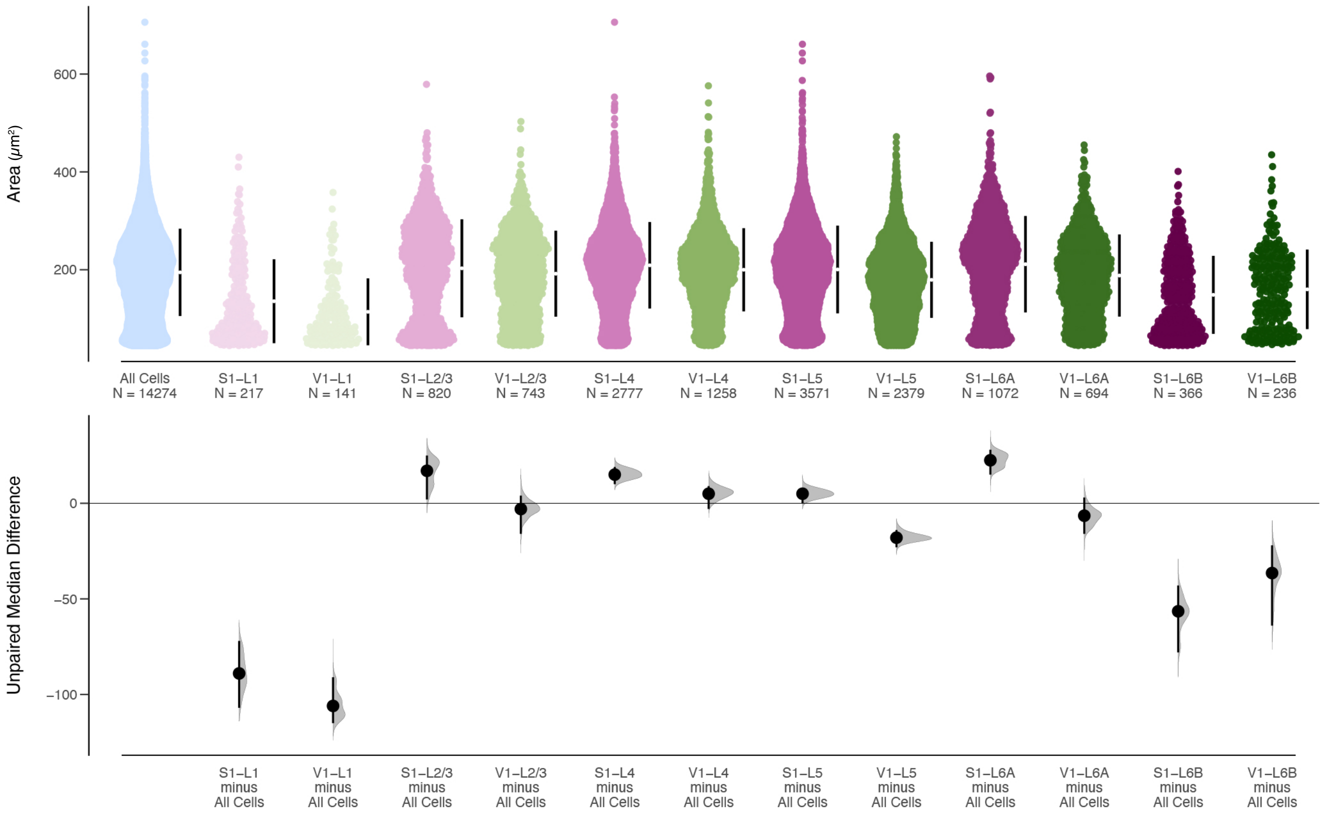

Supplement: Supplementary file 3 [file Data_Sheet_2.pdf]
