# Supplementary material for: A high-dimensional atlas of parvalbumin interneuron soma morphology in mouse visual and somatosensory cortex
Source: Front Neurosci. 2026 Jun 10;20:1848222. doi: 10.3389/fnins.2026.1848222 (PMC13290952; doi:10.3389/fnins.2026.1848222)

Supplementary Figure 4
Quantifying animal-specific distributions across clusters

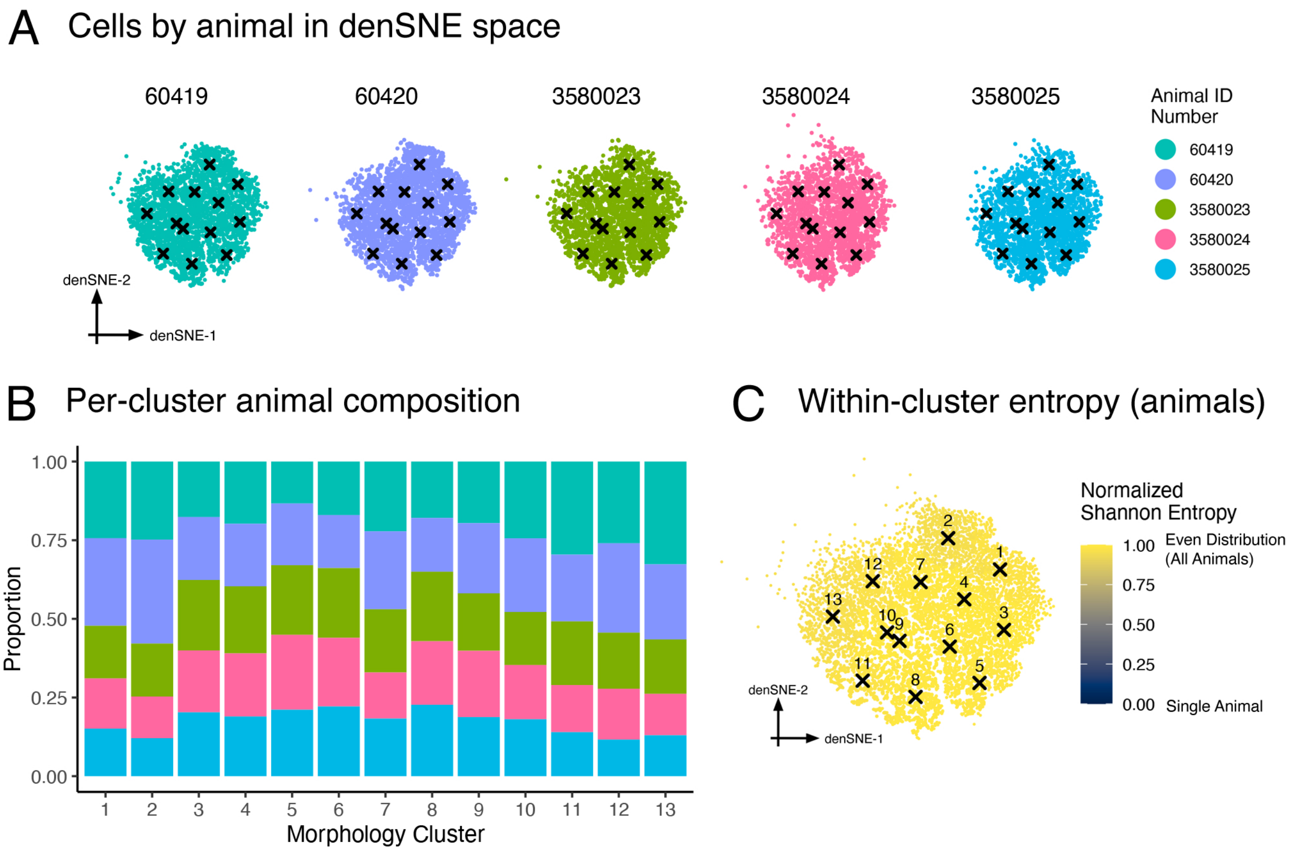

Supplementary Figure 5
Estimation Statistics on Composite Shape Properties

A Composite Circularity

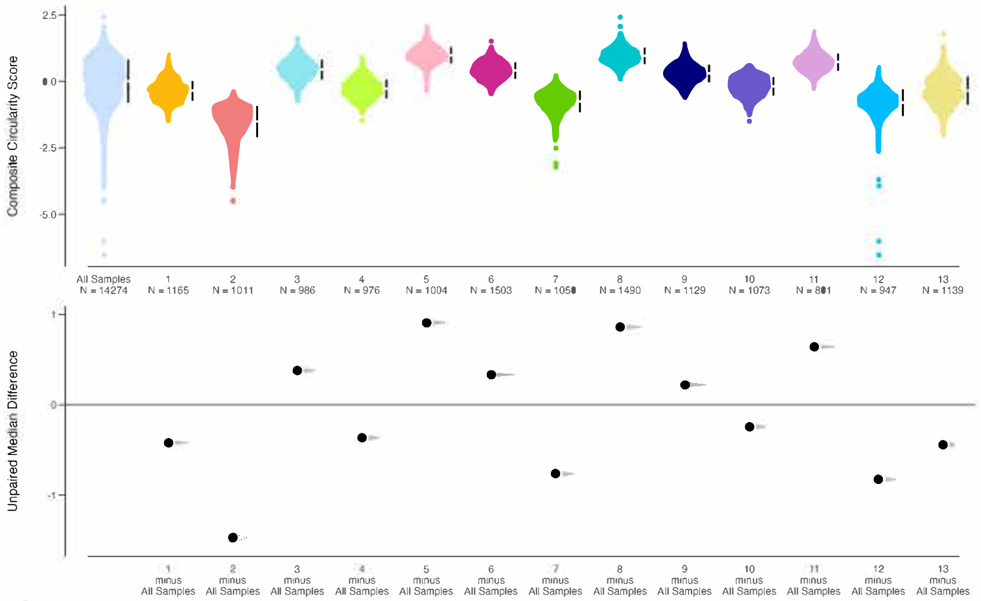

B Composite concavity

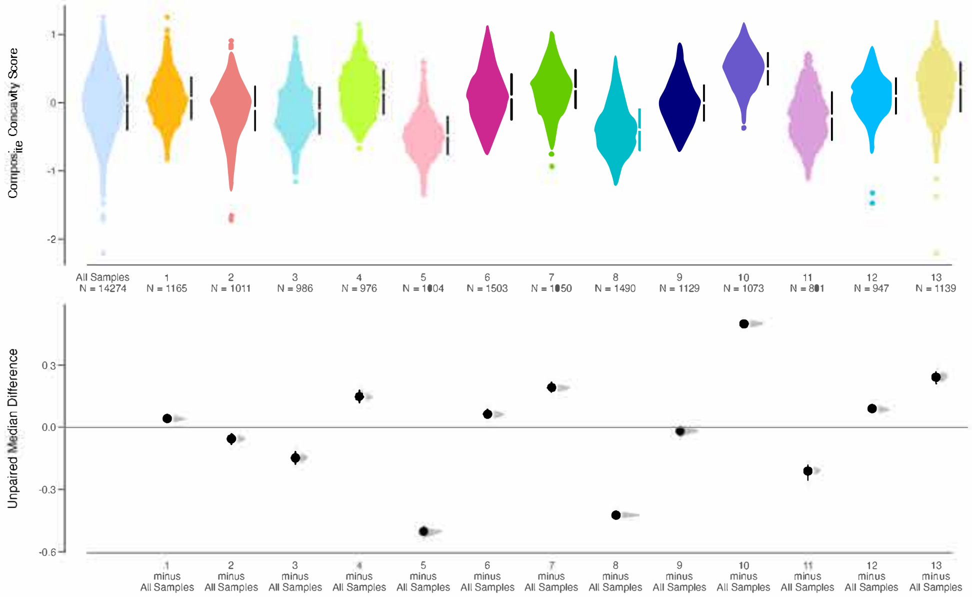

C Composite protrusion

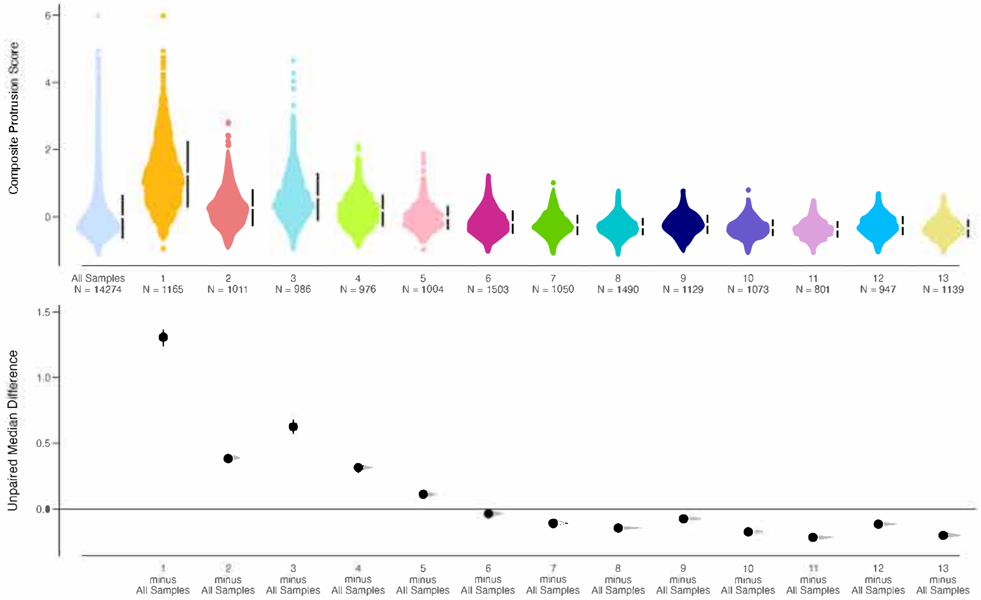

Supplementary Figure **6**
Area Biases of PV+ Morphology Clusters

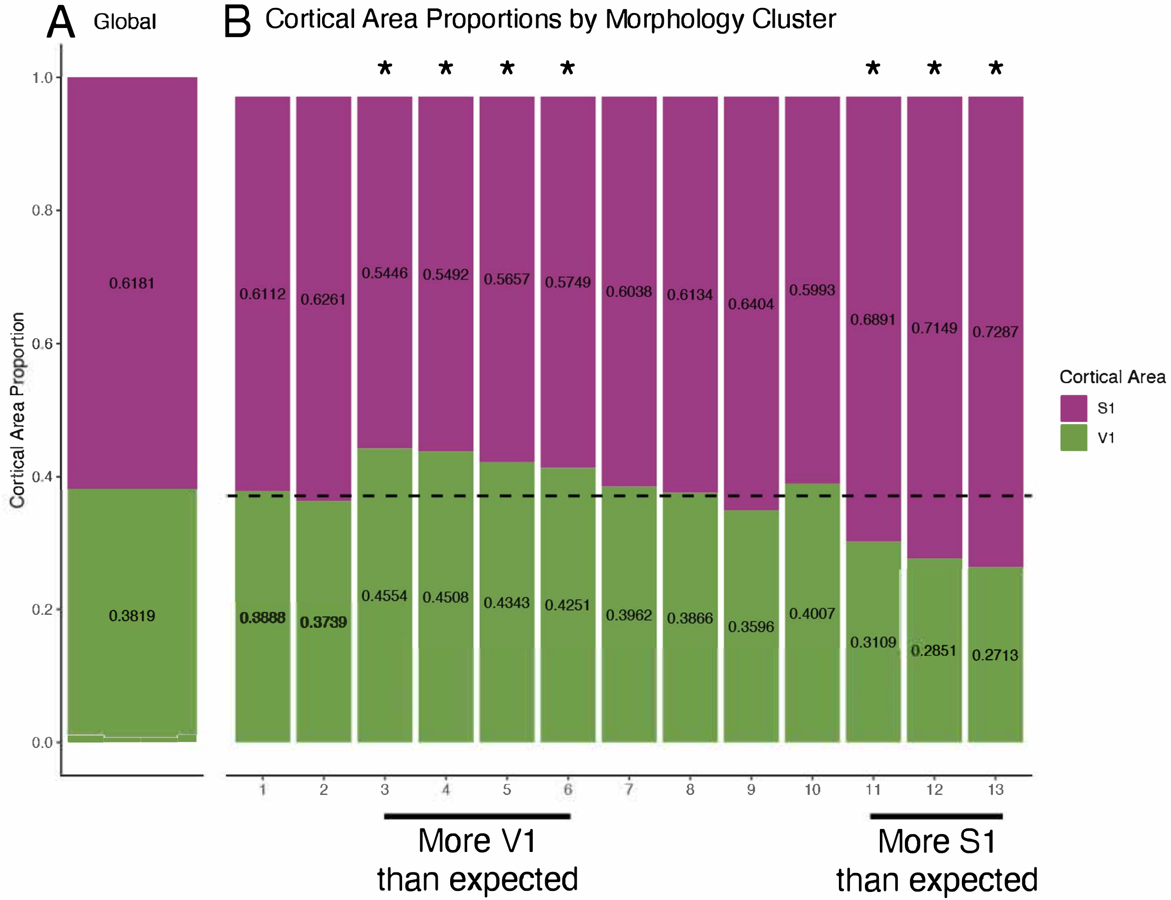

Supplement: Supplementary file 4 [file Data_Sheet_3.pdf]
