# Supplementary material for: A high-dimensional atlas of parvalbumin interneuron soma morphology in mouse visual and somatosensory cortex
Source: Front Neurosci. 2026 Jun 10;20:1848222. doi: 10.3389/fnins.2026.1848222 (PMC13290952; doi:10.3389/fnins.2026.1848222)
Supplement: Supplementary file 5 [file Data_Sheet_4.pdf]

# A Protocol for Cell Body Morphology Extraction and Quantification in CellProfiler

| | |
|---|---|
| **Category** | Experimental Procedures |
| **Author** | Maheshwar Panday |
| **Version** | 2 |

**Labels:** MP - Neuroanatomy

## Programmatic Image Retireval using AllenSDK

Whole section-images of in situ hybridization (ISH) can be downloaded programmatically using a jupyter notebook published by the Allen Institute for Brain Science, as part of the Allen software development kit (allensdk package in python).
The jupyter notebook can be found here : **https://allensdk.readthedocs.io/en/latest/_static/examples/nb/image_download.html**

- Note: expression images (the pre-processed images where cells are identified against a black background, colour-coded by their integrated optical densities must be downloaded manually from the Allen Brain Atlas: Mouse Brain Database : **https://mouse.brain-map.org/**
- Note : newer versions of python may not support older versions of core packages needed to run the allensdk suite of tools. To work around this, you can try creating a virtual environment with an older version of python (version 3.9.13 supports allensdk & dependencies) : **https://community.brain-map.org/t/programmatic-download-of-human-ish-issues-with-allensdk/4846**

## Loading Images in CellProfiler for Analysis

The first module at the top of all pipelines is the "Images" Module. This is where image samples are loaded for analysis. If all of your samples of expression images are stored in a single folder, the folder and be dragged and dropped into the image loading window. If you have image files and other file types in the same folders, you can filter files in your input file list to include only images.

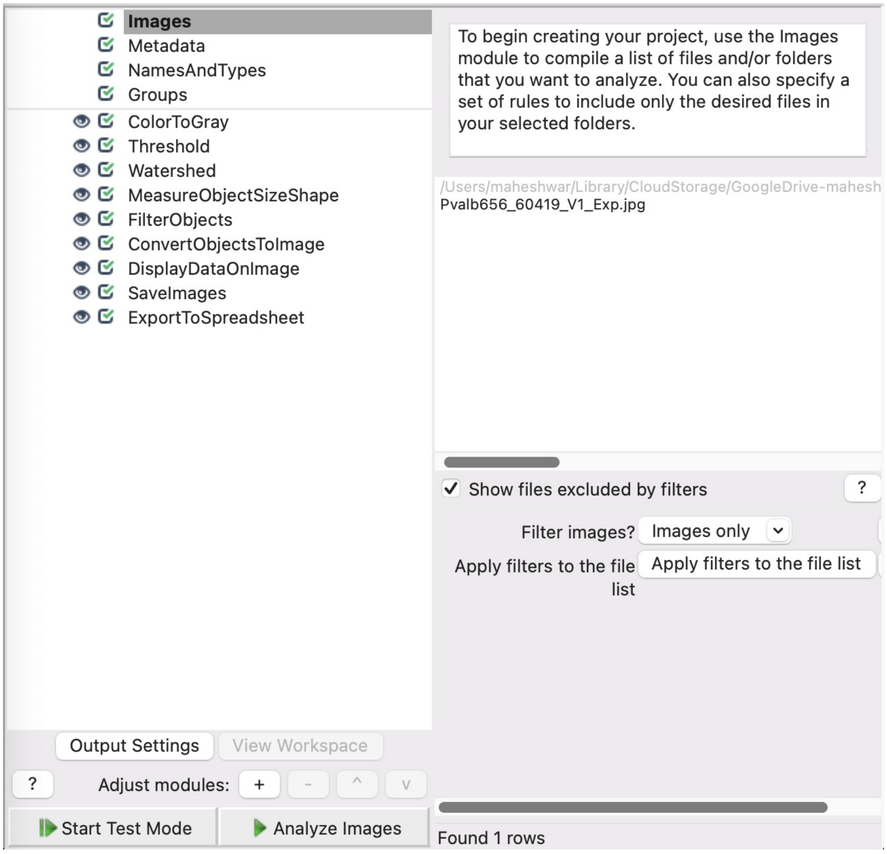

Fig.1 - Images module with a single sample image loaded in the input field.

## Extract and Organize Sample Metadata from Image Filenames

# Configuring the Metadata Module for Image Analysis

It is important to ensure your individual image samples have metadata written into the filenames. The naming system must be consistent to ensure smooth, consistent metadata extraction using CellProfiler.

1. Select "Yes" to extract metadata.
2. Indicate your Metadata are to be extracted from the file names
3. To parse filenames - provide a regular expression : this is the syntactic search pattern that CellProfiler will apply to individual filenames to extract metadata parameters accordingly.
4. Indicate that Metadata are to be extracted from all images.

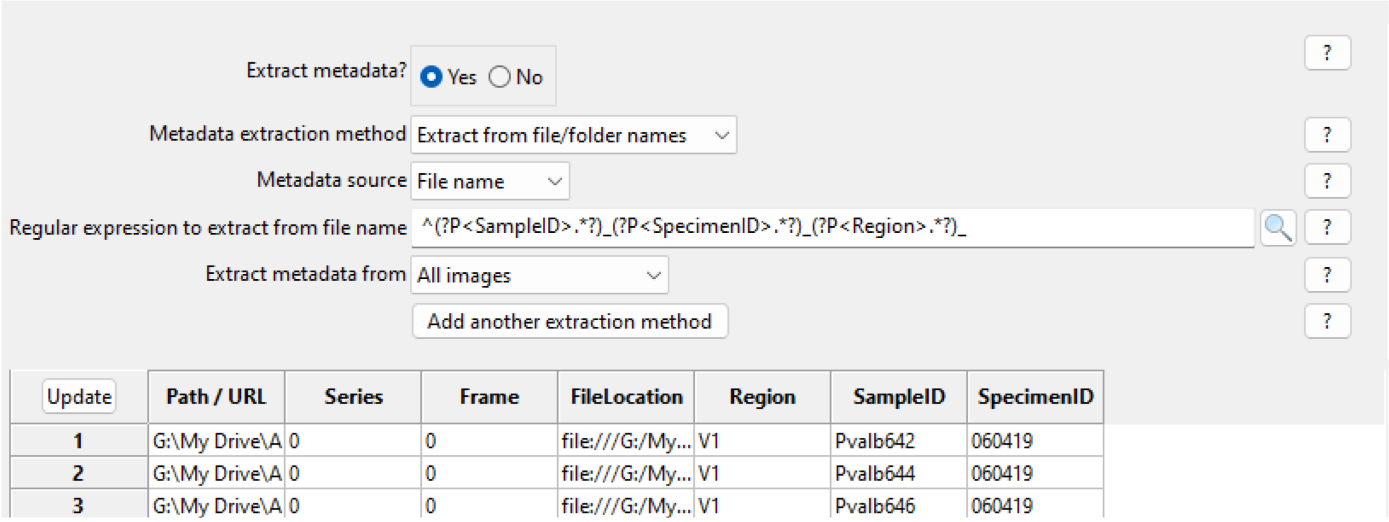

Figure 2 - Metadata Module Showing Metadata Extraction from File Names with a Regular Expression

## Example - Metadata Naming Conventions for Mouse Cortex Samples

Pvalb646_060419_V1_Exp.jpg

- Metadata_SampleID : The gene symbol (mouse convention) + the last three digits of the sectionID number as defined by the Allen Institute
- Metadata_SpecimenID : The unique number to identify the animal from which this set of sagittal sections was obtained (hyphens removed), note if IDs start with 0, the first 0 will be dropped in the dataframe returned from CellProfiler
- Metadata_Region : The anatomical region of interest (V1 or S1)

- Pvalb642_060419_V1_Exp.jpg
- Pvalb644_060419_V1_Exp.jpg
- Pvalb646_060419_V1_Exp.jpg
- Pvalb648_060419_V1_Exp.jpg
- Pvalb652_060419_V1_Exp.jpg

Example of 5 sample names for images being passed to analysis

### Configure Image Handling Rules in CellProfiler

## Process all expression images consistently with the NamesandTypes module

Image handling rules ensure all expression images are handled consistently with the same steps applied to all images. This module also allows you to assign a meaningful name to each image, which other modules will inherit from. As all images used and analysed with the steps in this protocol are expression images. We can apply a universal naming convention at this step so all expression images are processed and analysed in the same way.

1. Assign a name to all images

2. Select "No"' for Process as 3D

3. Select "Color Image" for theimage type

4. Assign a meaniingful name to these images ( your input images will be referred to this name as you configure the downstream pipeline).

5. Set the intensity range from the pixel bit depth.

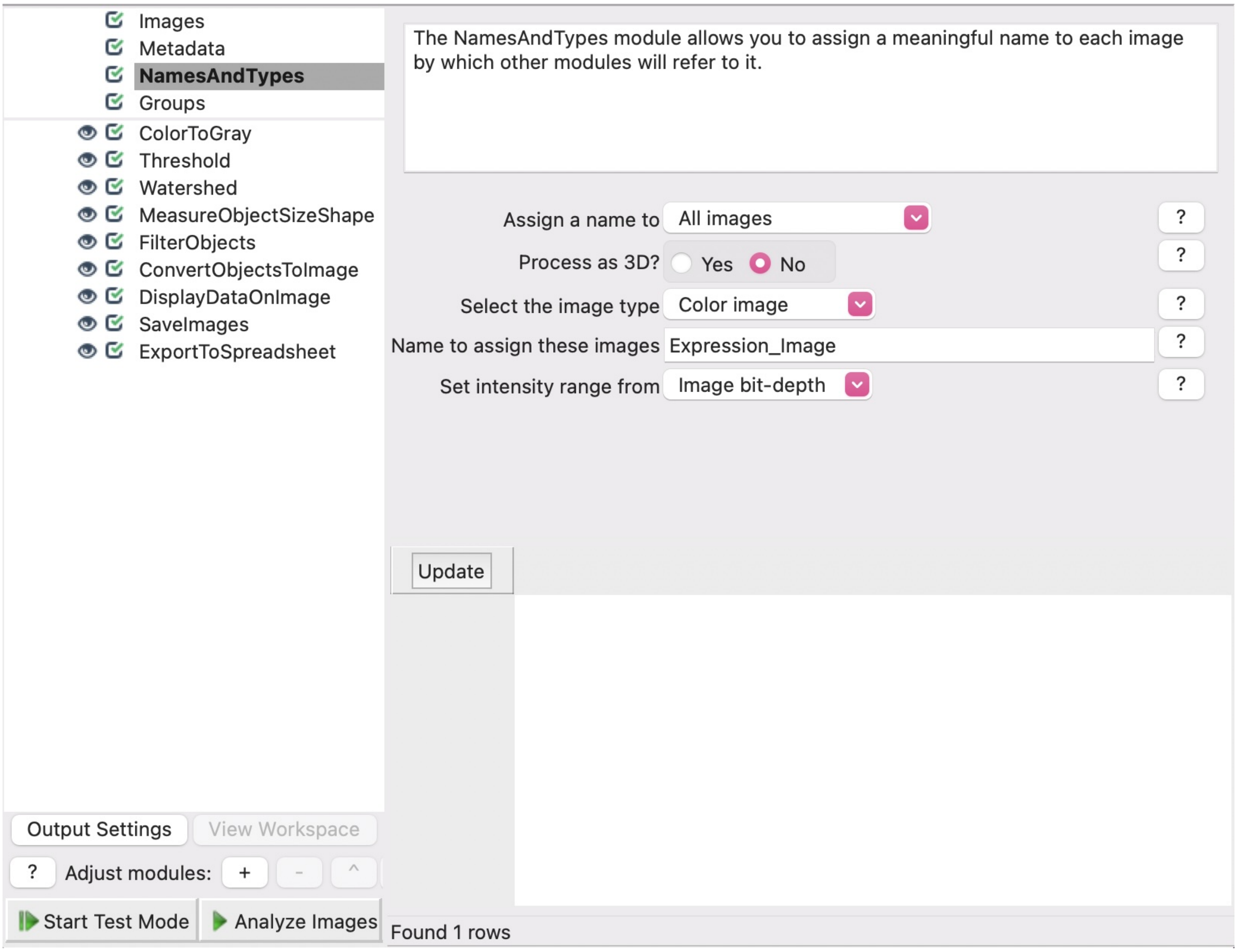


Fig. 3 - image handling rules configured in the names and types module

## Setting the Groups Module

The groups module optionally allows you to split your list of images into image subsets (groups) which will be processed independently of each. Because all expression images are pre-processed using consistent steps regardless of anatomical location, grouping by anatomical structure is not strictly required at this step. Grouping by anatomical structure can be achieved using the metadata in downstream data analysis.

1. Answer "No" to the question "Do you want to group your images"

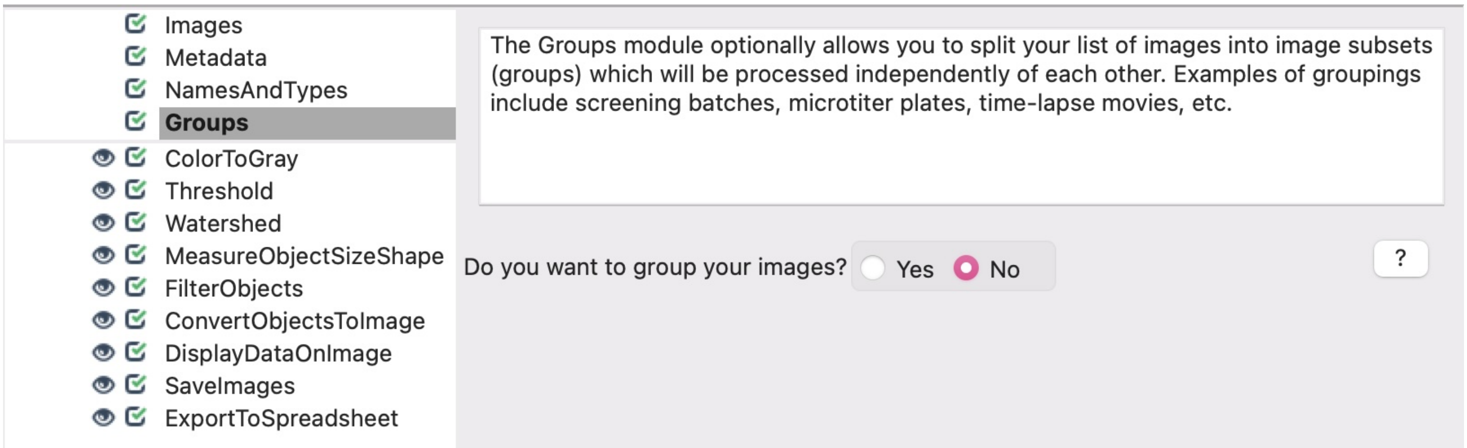


Fig. 4 -Inside of groups module - select "no" to process and all images together.

## Image Processing : Expression→Grayscale→Threshold→Segmentation

This first collection of steps will process the RGB Expression Image for Analysis. This series of  image processing modules ensures that all images are binary (white signal on a black background), and that individual objects are isolated, physically separate from adjacent objects in the image before analysing the morphologies of individual objects. At this point in the protocol, true cells and particles are present in the images. To simplify terminology, we refer to all objects in processed images as objects.

## Convert RGB Expression Images to Grayscale

The first step in this pipeline is to convert the expression image from RGB to gray levels. Using the ColorToGray Module.
This module converts the input RGB Expression Map images into gray levels for downstream analysis. Note that grayscale images are required for thresholding, which is necessary for object segmentation by Watershed.

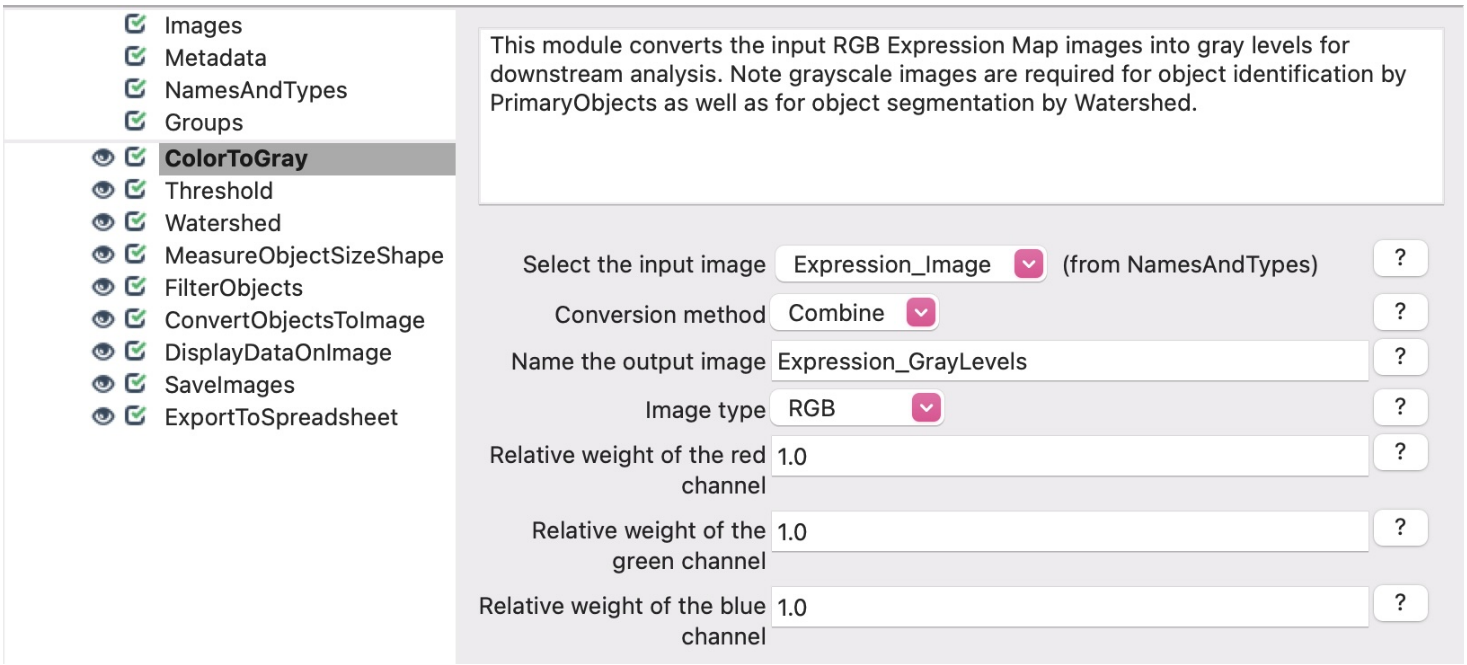

Figure 5 - settings configurable in the ColorToGray Module

1. Select the ColorToGray Module

2. Select the input image (Expression_Image from the NamesandTypes Module) - all expression images are being processed

3. Select the conversion method as combine (this merges the red, green and blue channels together into a single grayscale image)

4. The image type is RGB

5. Set the relative weights of the red blue and green channels (for combination) - set them all as equal to 1.0

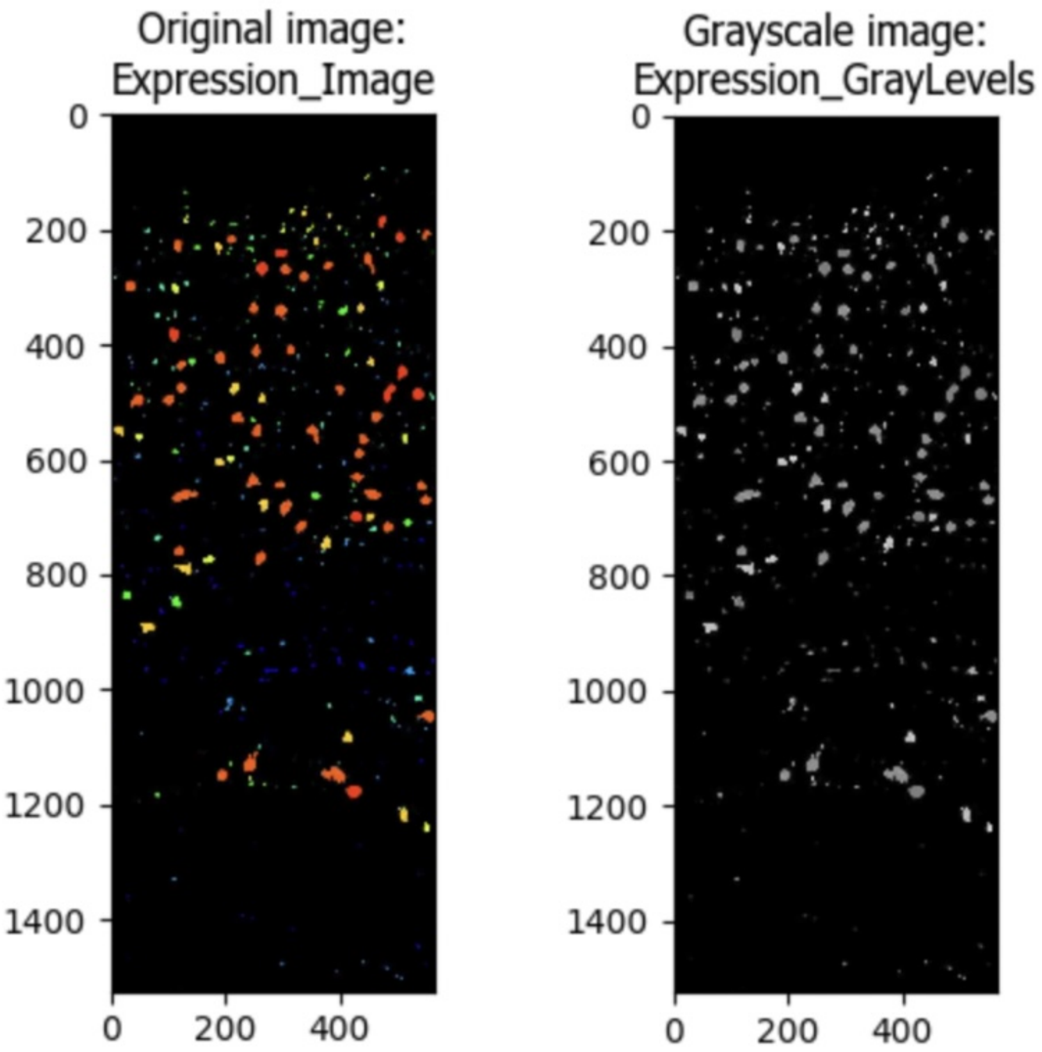


Fig. 6 - output from ColorToGray converts the RGB Expression Image to Grayscale

## Apply Thresholding to Binarize the Grayscale Image

The next image processing step takes the grayscale image, and with the Threshold module, converts it to a binary image. This step is necessary for the watershed module which performs cell segmentation. Because the Allen Institute processed In Situ Hybridization images to produce an expression image with a uniform black background, with particles pseudocoloured by their integrated densities, we apply the default thresholding parameters. A variable background in ISH images might require additional parameter tuning, but the default settings are sufficient for thresholding the expression image from gray levels.

The Threshold module inherits the grayscale image from the previous ColorToGray module as its input and outputs a binarised image (white signal on a black background).

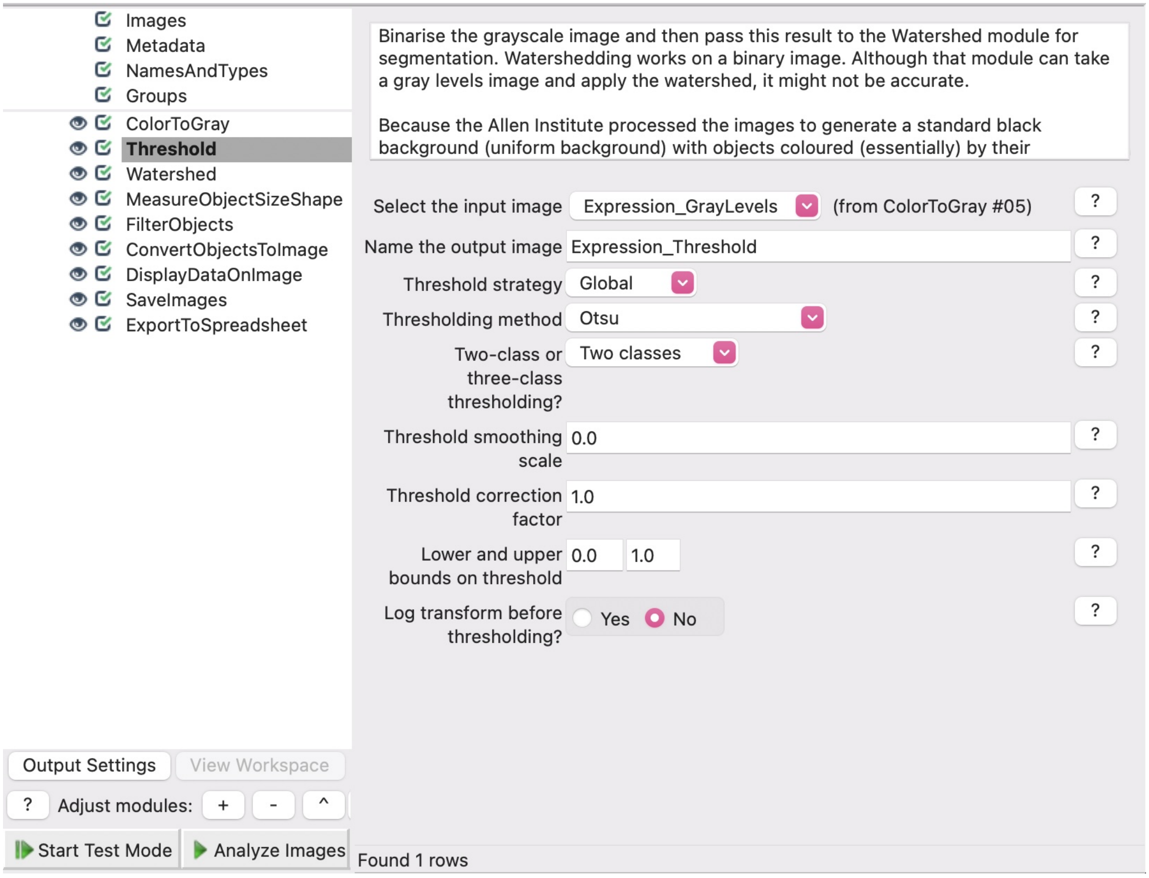


Fig. 7 - parameters configurable in the Threshold module. This module inherits the grayscale image and outputs a binary image of white signal on a black background.

1. Select the input image - the grayscale image produced in the ColorToGray module

2. Assign a name to your output image - this will the image name that the subsequent watershed module inherits as its input.

3. Select a global thresholding strategy - which is optimal for samples with uniform background

4. Select Otsu's method for thresholding - the algorithm that works best in global two-class thresholding cases

5. Select two-class thresholding - there are only two cases : the objects (foreground) and the background

6. Set a threshold smoothing scale of 0. This does not smooth the image.

7. Set a threshold correction factor of 1. This value does not apply a correction to the thresholding performed. A correction factor between 0,1 makes for more permissive thresholding, and correction factors greater than 1 make thresholding more stringent.

8. Do not log transform the image intensity values before thresholding.

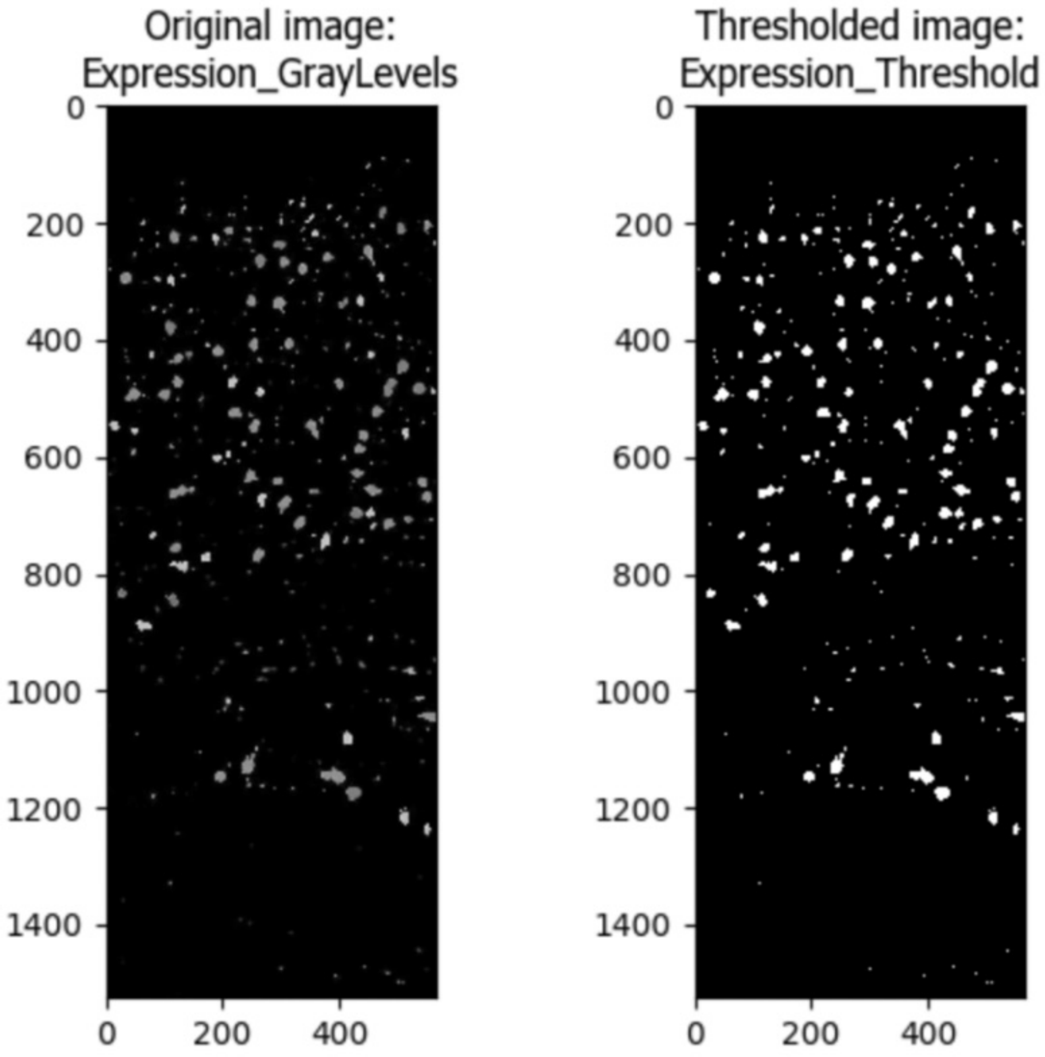


Fig. 8 - Thresholding image output as two classes: objects in white and background in black.

## Segment adjacent objects with the Watershed module

The watershed module is the final image processing step before proceeding to morphology extraction. This step applies a distance-based watershed segmentation algorithm to separate adjacent objects that appear to be touching in the image. This module inherits the binary image and outputs a segmented binary image.

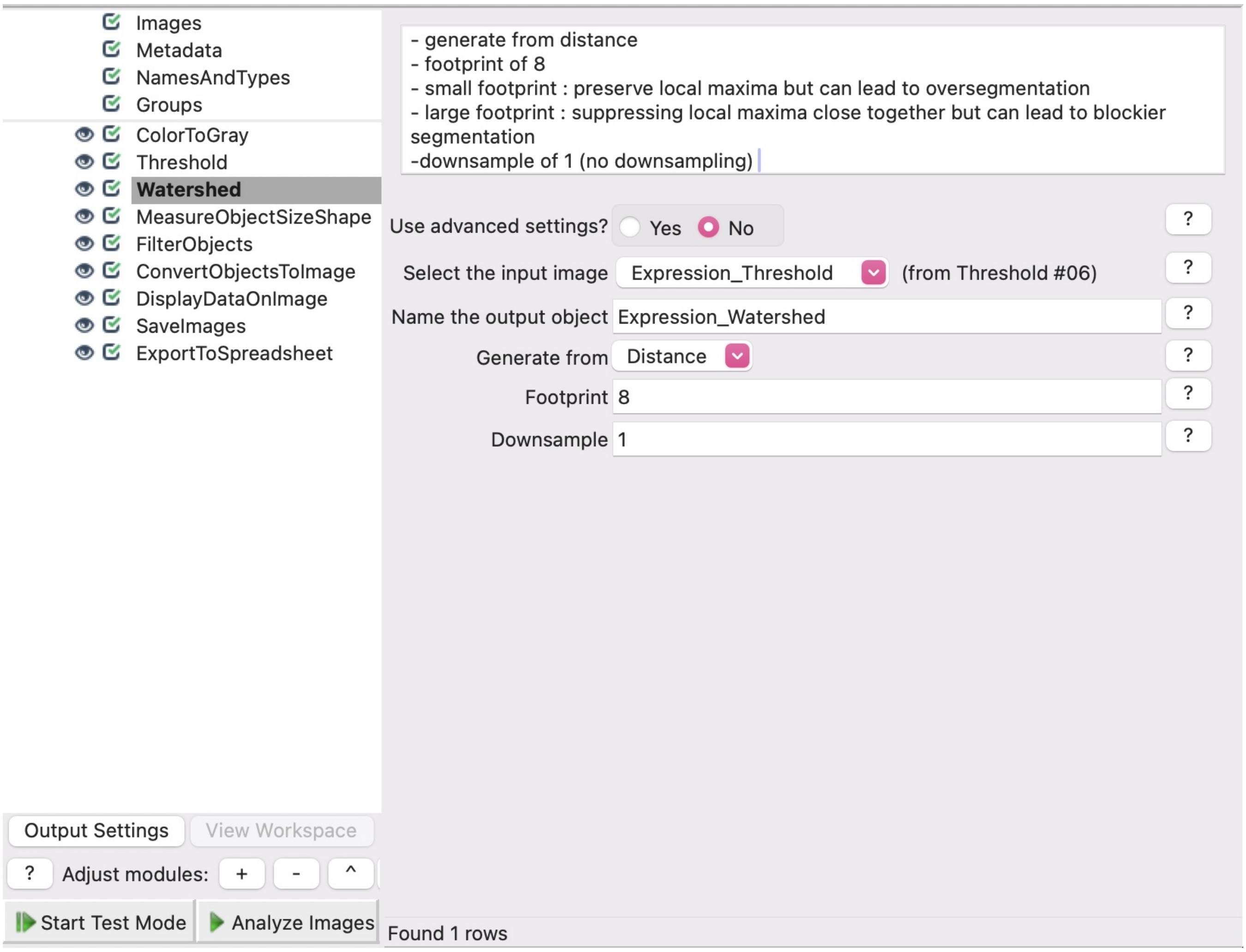


Fig.9 - parameters configurable for the watershed module.

1. Do not use advanced settings for ISH Expression images

2. Select the input image - this module inherits the binarized image generated in the Threshold module and outputs the segmented binary image called Expression_Watershed

3. Generate the segmentation from distances. For this segmentation, images must be binary. Because of the appearance of objects in the original expression image, this approach is generally highly suitable.

4. Select a footprint of 8

5. Set the downsampling parameter to 1 - this is the value that corresponds to no downsampling. Images maintain their full resolution throughout the pipeline.

Importantly, when previewing images output from the watershed module, outputs are pseudocoloured to visualise the segmentation produced by the watershed module. Obects are pseudocoloured such that no two adjacent objects are the same colour. This colour scheme has no bearing on the size of the cells, as was the case with the input expression images at the start of this image analysis pipeline.

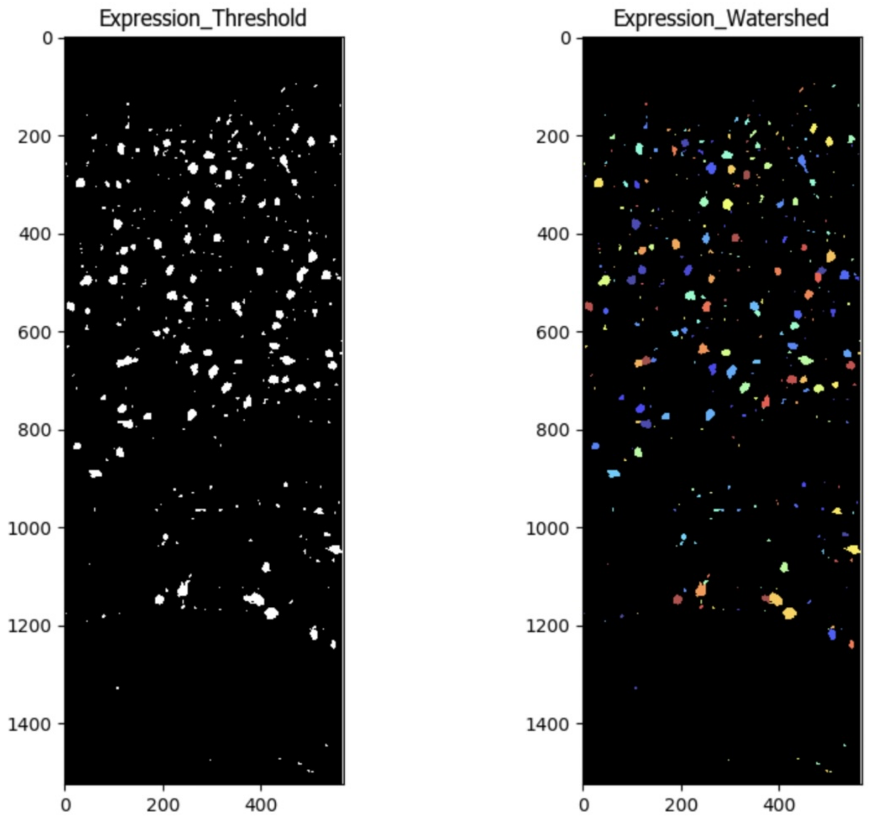


Fig. 10 - watershed output pseudocoloured to inspect the segmentation

## Morphology Extraction : MeasureObjectSizeShape

Now that the images are processed, the next step is to extract high-dimensional morphology data. This step is applied to all objects visible in all images. This includes true cells as well as particles and debris. Downstream data filtration steps will be necessary to clean these data prior to analysis.

1. Select the object set from the watershed module - this measurement module will inherit the watershed objects for quantification

2. Select yes to calculating Zernike Features - these are a series of shape moments that quantify additional shape properties such as circularity, elongation, symmetry and radial symmetrical arrangements of protrusions around the cell body.

3. Select yes to calculating the advanced features - these are additional shape moments that describe translation and rotation-invariant shape properties that complement geometric shape properties such as circularity, and abstract shape properties extracted from Zernike moments.

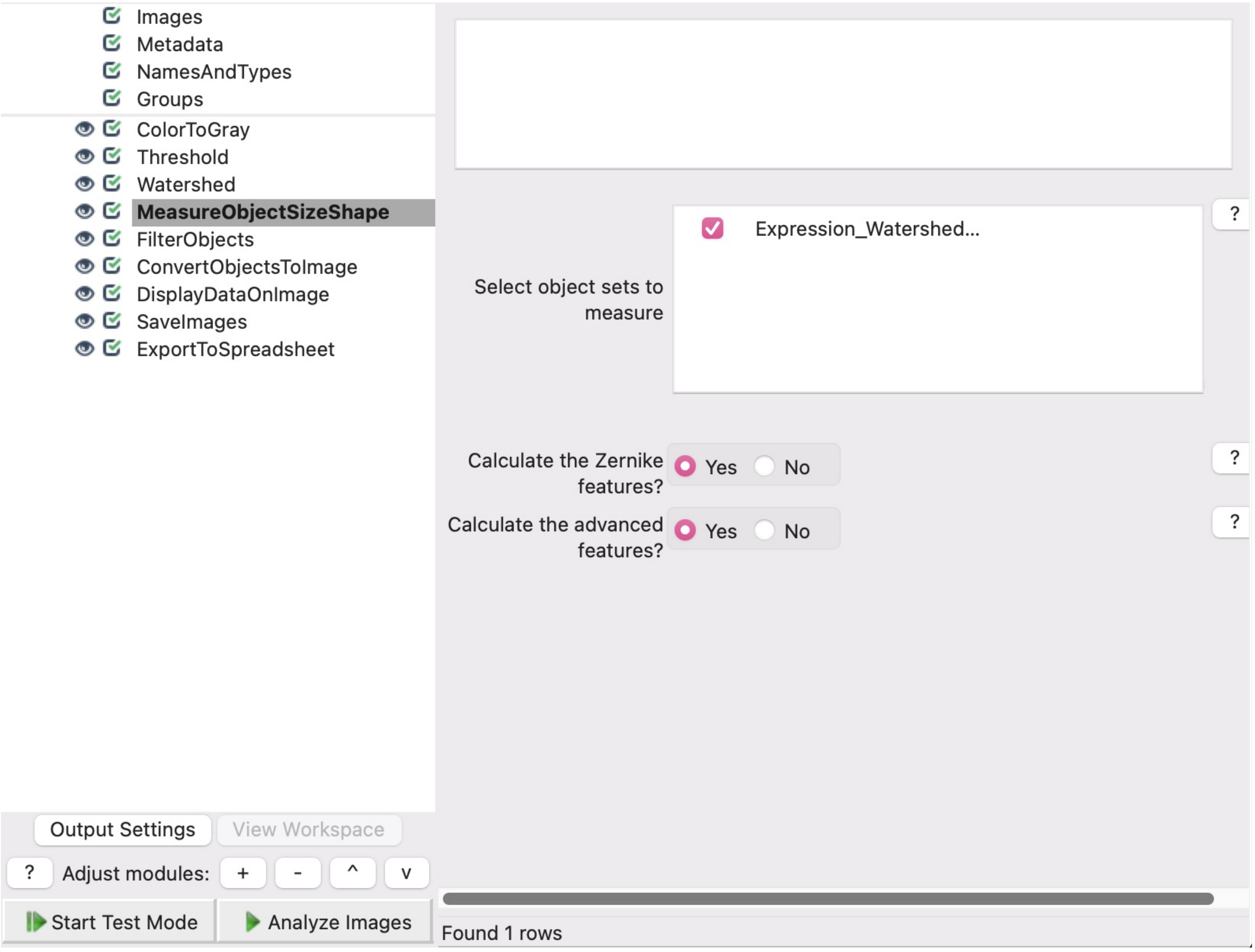


Fig. 11 - settings configurable in the MeasureObjectSizeShapeModule

## Test Filtration Values using the FilterObjects Module

The filter objects module lets you visually inspect the filtration. The filtered results are not exported, the complete unfiltered data are output as a spreadsheet. But this module is helpful to test which parameters work best before tuning the parameters more finely. For PV+ cells, cells are filtered by size and circularity ranges consistent with literature values (Kooijmans et al., 2020).

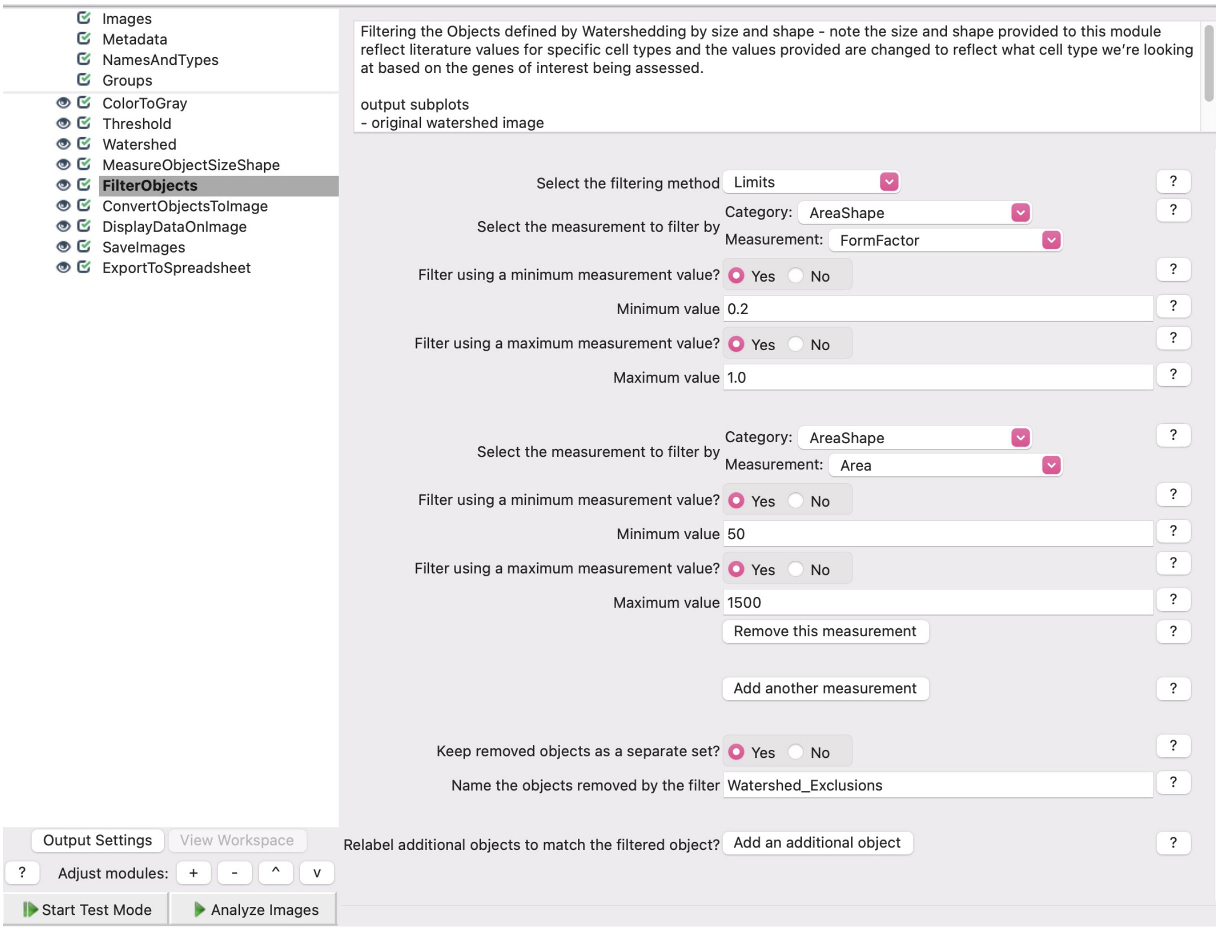

Fig. 12 - Settings configurable in the FilterObjects Module. Size and FormFactor(circularity) are used in the protocol

1. Select limits as the filtration method - values are included if they fall within the target range

2. Configure the first filter to include cells with a FormFactor (Circularity) range minimum value = 0.2 and maximum value = 1.0

3. Configure a second filter to include cells with an Area (Size) range minimum value = 50$\mu m^2$ and maximum value = 1500 $\mu m^2$. Note because CellProfiler works in pixels it is agnostic to units. Allen Institute Mouse Brain Expression ISH maps have a resolution of 1 pixel = 1 µm.

4. Depending on your cell type of interest, the filtration ranges and the morphological values used in filtration may vary. These can be selected and tuned by the user as needed.

5. Keep removed objects as a separate set? Select "Yes" (this helps in inspecting which objects pass filtration, so you can tune the parameters accordingly)

6. Assign a name for the objects removed by the filter. We call it "Watershed_Exclusions"

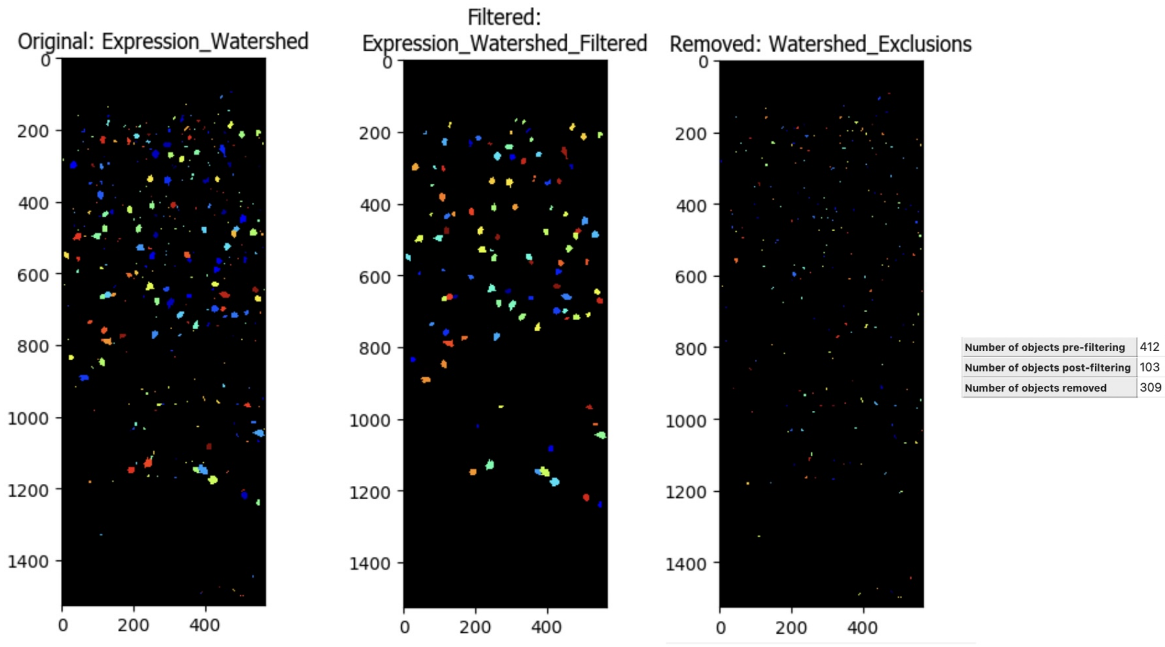


| | |
|---|---|
| Number of objects pre-filtering | 412 |
| Number of objects post-filtering | 103 |
| Number of objects removed | 309 |

Fig. 13 - Object filtration module results. Left input expression watershed image. Middle: watershed objects that pass filtration. Right : Watershed objects that are excluded following filtration. The table to the right shows the number of objects included and excluded by filtration.

## Visualise Segmentation and Filtration Results on Original Expression Images

You can display the results of the segmentation and filtration results in the display data on images module. This serves an inspection step to verify the quality of both the object segmentation and filtration. By displaying these results on the original expression image, you can determine whether/how to tune your segmentation and filtration parameters if you notice either of those steps are too permissive or too restrictive.

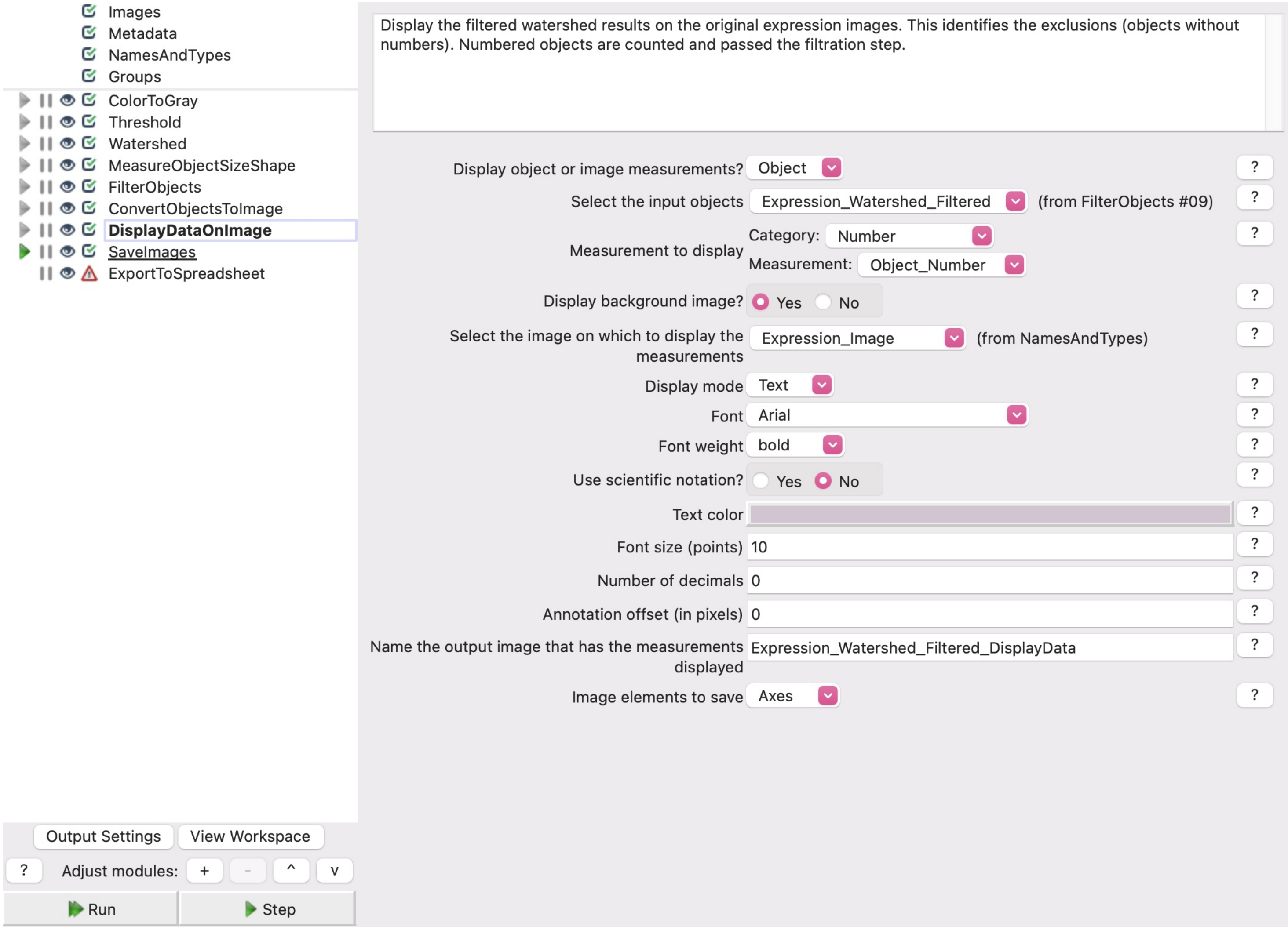


Fig. 13 - Settings configurable in the Display Data On Images Module

1. Display Object or Image Measurement ? : Select "Object"

2. Select the input objects : you can pass either the Expression_Watershed (unfiltered), Expression_Watershed_Filtered (pass filtration), Expression_Watershed_Exclusions (objects that get filtered out at the filter objects step).

3. Measurement to Display - Category = "Number", and Measurement = "Object_Number". This will display the object ID number on each object that passes the filtration and segmentation steps.

4. Display background image? : Select "Yes"

5. Select the image on which to display the measurements . Select "Expression_Image" this is the original expression images that are inputs to the images module

6. Display mode - select "Text"

7. Font - "Arial"

8. Font weight - "Bold"

9. Use Scientific Notation? Select "no"

10. Select a text colour - this is the colour used to display the text annotation on your image

11. font size (points) - select 10

12. Number of

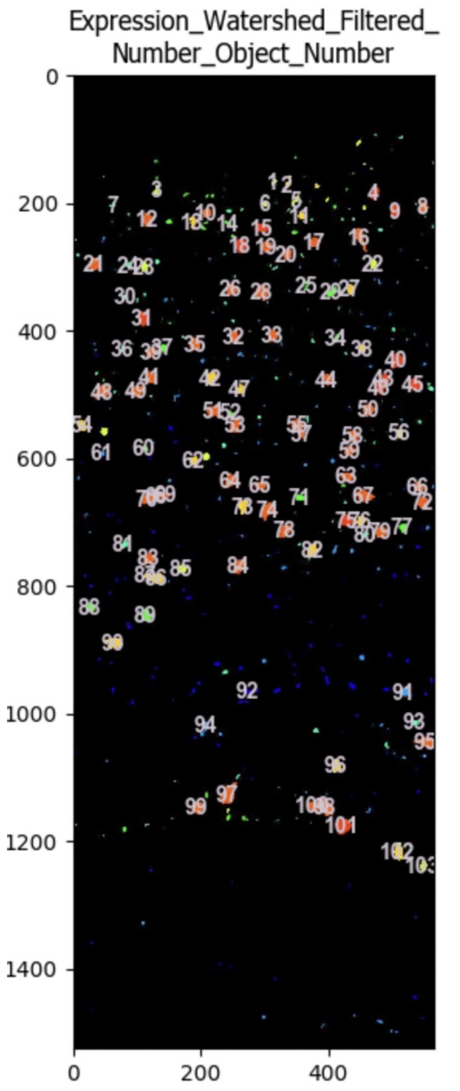


Fig. 14 - filtered objects displayed on the input expression image with the object numbers used to identify individual objects

## Saving and Exporting Annotated Image Maps and the Morphology Data Spreadsheet

# SaveImages : saving a copy of the annotated expression images

Use the SaveImages module to save a copy of the annotated expression images.

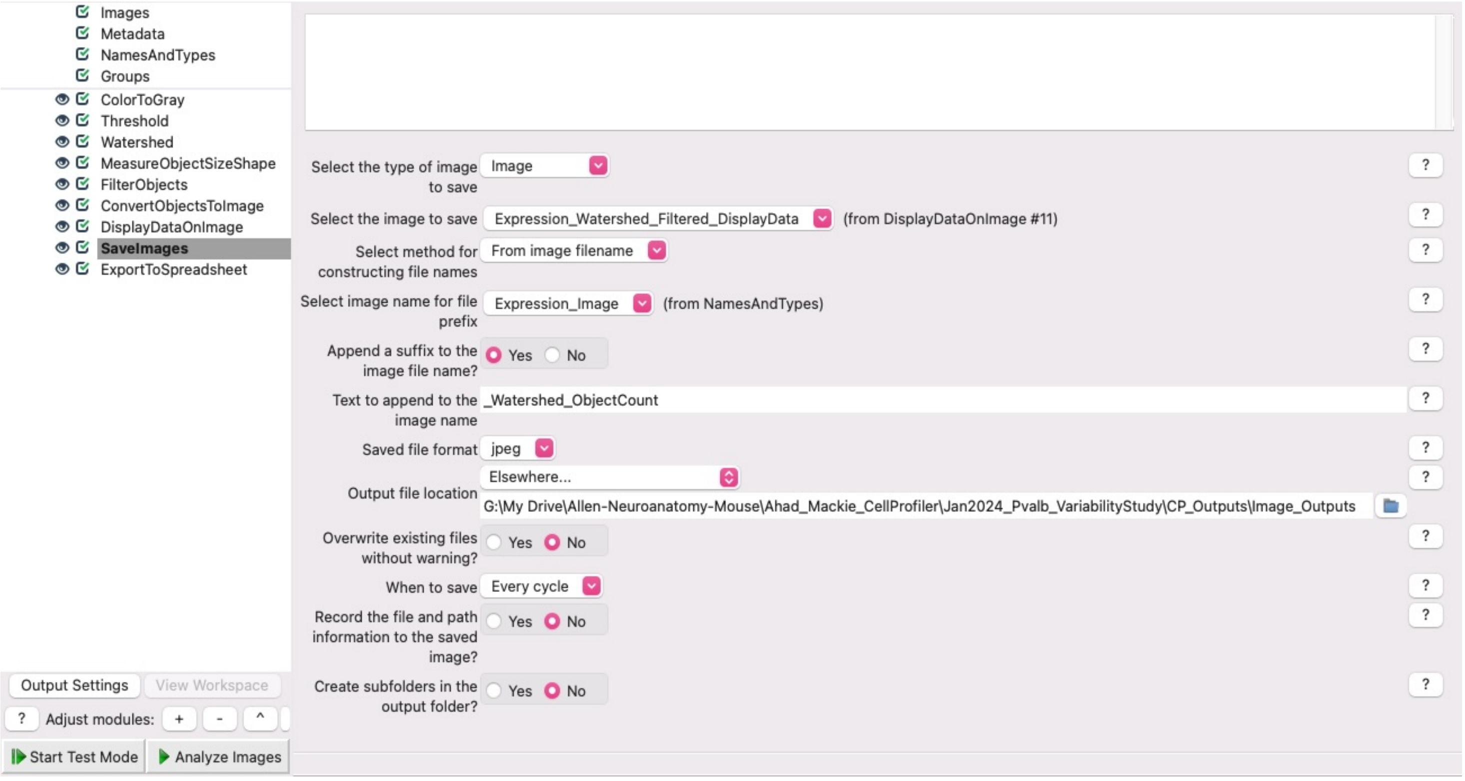


Fig. 15 - settings configurable in the SaveImages Module

1. Select the type of image to save : select "image"

2. select the image to save : select Expression_Watershed_Filtered_DisplayData (this is annotated expression image that has the filtered objects identified)

3. Select method for constructing file names "select "from image filename" (this will construct an output filename that inherits from the input filename)

4. Select image name for the file prefix : set a suitable prefix for your experiment "Expression_Image"

5. Append a suffix to the image file name ? Select "Yes" and set the text to append to the image name as related to your data

6. Save file format - select "jpeg"

7. Output file location : select "Elsewhere" and specify the file path to the destination folders

8. Overwrite files without warning? Select "No"

9. When to save - select "Every cycle" (each image that gets processed has a saved annotation output.

10. Record the file and path information to the saved image? Select "No"

11. Create subfolders inthe output folder? Select "No"

## ExportToSpreadsheet : saving the extracted cell morphology data

N.B. in test mode (as shown in the figure below, spreadsheets will not save, this module only runs when analysing images outside of test mode).

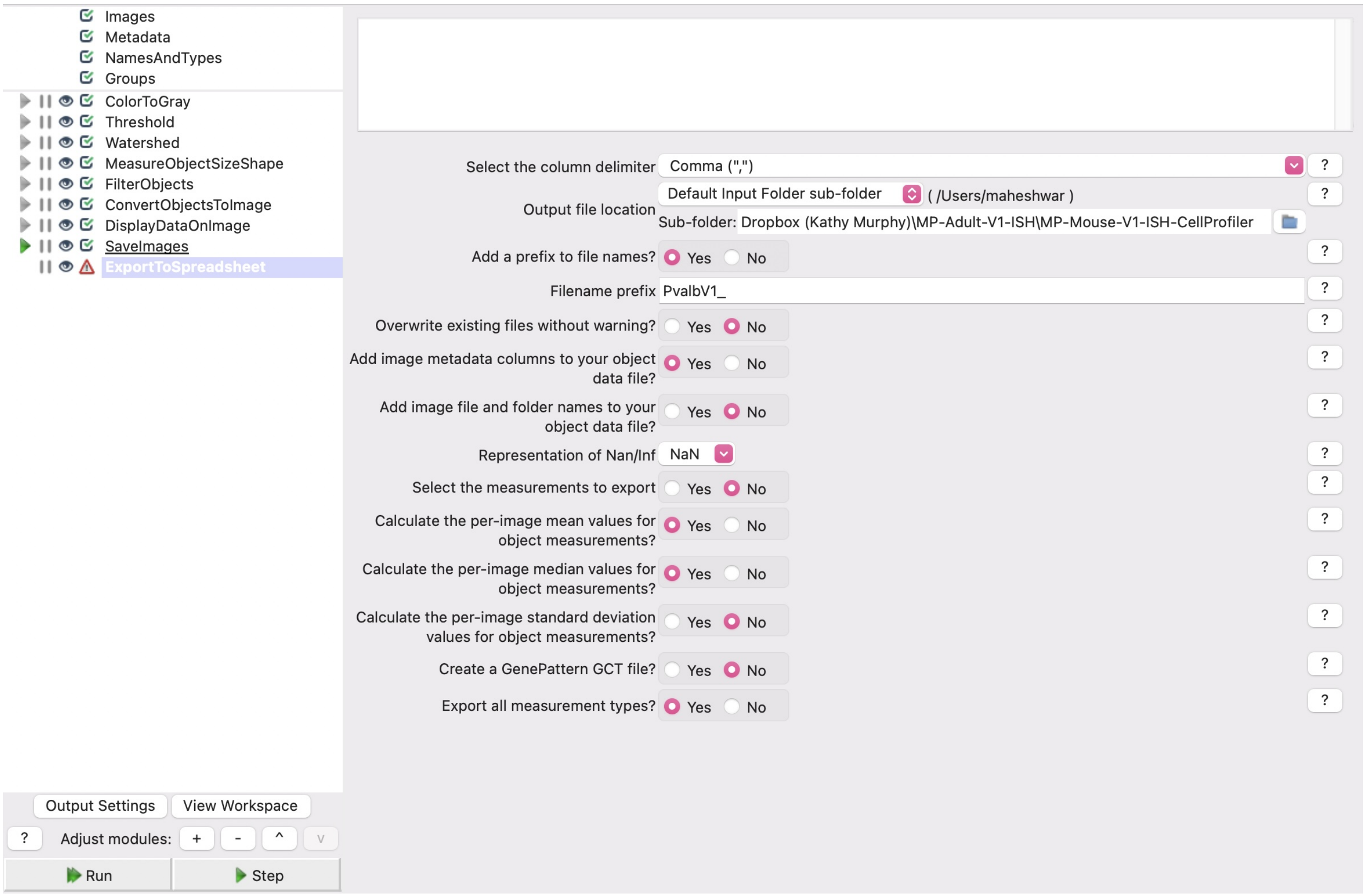


Fig. 16 - settings configurable in the ExportToSpreadsheet module

1. Select the column delimiter : "Comma"
2. Output file location - specify the path to your destination folder
3. Add a prefix to the filenames : select "Yes" and specify the filename prefix. Specify the text relevant to your data.
4. Overwrite existing files without warning? Select "No"
5. Add image metadata columns to your data file? Select "Yes
6. Add image file and folder names to your object data file? Select "No"
7. Representation of Nan/Inf: select NaN
8. Select the measurements to export : select "No" so that all measurements get exported to the measurement csv file
9. Calculate the per-image mean values for object measurements ? Select "Yes"
10. Calculate the per-image median values for object measurements ? Select "Yes"
11. Calculate the per-image standard deviations for object measurements? Select "No"
12. generate a GenePattern GCT file? Select "No"
13. Export all measurement types? Select "Yes" (Note if you want to specify specific measurements to be exported, or set a nomenclature other than default, select "No").

## References


Ebsy Jaimon, Suzanne R Pfeffer 2023. A computational pipeline to quantify perinuclear lysosomes in fibroblasts using CellProfiler. **protocols.io**https://dx.doi.org/10.17504/protocols.io.81wgbxrw3lpk/v1

Stirling DR, Swain-Bowden MJ, Lucas AM, Carpenter AE, Cimini BA, Goodman A (2021). CellProfiler 4: improvements in speed, utility and

usability. BMC Bioinformatics, 22 (1), 433. . PMID: 34507520 PMCID: PMC8431850

## Attachments

No file attachments

*This procedure was originally created by **Negeen Halabian***

Powered by SciSure
